# Bridging Traditional Wisdom and Evidence-Based Pharmaceutics: Comprehensive Specification and Biological Activity of the Wannachawee Recipe for Psoriasis

**DOI:** 10.3390/plants15091344

**Published:** 2026-04-28

**Authors:** Supreeya Tantipat, Wannaree Charoensup, Kongkiat Trisuwan, Phraepakaporn Kunnaja, Seewaboon Sireeratawong, Surapol Natakankitkul, Surasak Imiam, Apinya Rachkeeree, Ratchuporn Suksathan, Sunee Chansakaow

**Affiliations:** 1 Department of Pharmaceutical Sciences, Faculty of Pharmacy, Chiang Mai University, Chiang Mai 50200, Thailand; yayeetantipat@gmail.com (S.T.); wannareecharoensup@gmail.com (W.C.); 2 Department of Chemistry, Faculty of Science, Chiang Mai University, Chiang Mai 50200, Thailand; kongkiat.t@cmu.ac.th; 3 Research Laboratory on Advanced Materials for Sensor and Biosensor Innovation, Materials Science Research Center, Center of Excellence for Innovation in Chemistry, Faculty of Science, Chiang Mai University, Chiang Mai 50200, Thailand; 4 Department of Medical Technology, Faculty of Associated Medical Sciences, Chiang Mai University, Chiang Mai 50200, Thailand; phraepakaporn.k@cmu.ac.th; 5 Department of Pharmacology, Faculty of Medicine, Chiang Mai University, Chiang Mai 50200, Thailand; seewaboon@gmail.com; 6 Clinical Research Center for Food and Herbal Product Trials and Development (CR-FAH), Faculty of Medicine, Chiang Mai University, Chiang Mai 50200, Thailand; 7 Division of Herbal Innovation for Sustainable Quality of Life, Graduate School, Payap University, Chiang Mai 50000, Thailand; surapol_n@payap.ac.th; 8 Department of Thai Traditional Medicine, Phrapokklao Hospital, Chanthaburi 22000, Thailand; surasak.alexx@gmail.com; 9 Queen Sirikit Botanic Garden, The Botanical Garden Organization, Chiang Mai 50180, Thailand; chordata_rose@hotmail.co.th (A.R.); r_spanuchat@yahoo.com (R.S.)

**Keywords:** Wannachawee recipe (WCR), Thai traditional medicine, psoriasis, quality control, compact mass spectrometry (CMS), high-performance liquid chromatography (HPLC), herbal specification

## Abstract

The Wannachawee Recipe (WCR) is a traditional Thai herbal formulation with a clinical history of use in psoriasis. An observational study conducted at Prapokklao Hospital reported that 93% of psoriasis patients showed good clinical responses. However, the absence of standardized quality control parameters remains a critical barrier to its pharmaceutical reproducibility, safety, and integration into mainstream clinical practice. This study established robust quality specifications and a phytochemical profiling for WCR, in accordance with the Thai Herbal Pharmacopoeia (THP) guidelines, to support its development from traditional use to a standardized therapeutic agent. A multimodal analytical approach was employed, integrating microscopic characterization, physicochemical evaluation, and advanced instrumental techniques. Phytochemical characterization was conducted using High-Performance Liquid Chromatography (HPLC) fingerprinting and Compact Mass Spectrometry (CMS). A validated HPLC method was developed to quantify *trans*-*p*-coumaryl alcohol, a key bioactive marker. Anti-inflammatory activity was further assessed by measuring inhibition of nitric oxide production. Physicochemical analysis established rigorous benchmarks, including ethanol-soluble extractive (8.73 ± 0.15% *w*/*w*), water-soluble extractive (18.89 ± 0.09% *w*/*w*), and loss on drying (<10%), which ensure long-term stability and microbial safety. CMS analysis successfully identified key chemical constituents, including alpha-amyrin, stemone, protocatechuic acid, and *trans*-*p*-coumaryl alcohol. HPLC fingerprinting demonstrated high batch-to-batch consistency, while quantitative analysis determined a *trans*-*p*-coumaryl alcohol content of 8.77 mg/g extract. Critically, biological evaluation showed that WCR exhibited potent anti-inflammatory activity by inhibiting nitric oxide production, with a superior inhibitory effect compared with the reference drug indomethacin. This study provides a preliminary scientific framework for the standardization of WCR. It defines precise quality specifications and a potential bioactive marker, establishing the rigor needed for regulatory certification and industrial production. This work connects traditional Thai medicine with evidence-based pharmaceutics, positioning WCR as a promising therapy for psoriasis.

## 1. Introduction

Psoriasis is a chronic, immune-mediated inflammatory skin disorder characterized by erythematous, pruritic, and scaly plaques [1]. While systemic biological therapies have revolutionized management for moderate-to-severe cases, their long-term use is often limited by high costs and the risk of severe adverse effects, including prolonged immunosuppression and increased susceptibility to malignancies. Consequently, there is a global imperative to develop novel antipsoriatic agents that offer a favorable safety-to-efficacy ratio and improved cost-effectiveness. Traditional medicine remains a vital therapeutic resource, serving as a primary modality for billions worldwide [1]. In Thailand, the Wannachawee Recipe (WCR), a polyherbal formulation, has been clinically used to manage psoriasis at the Thai Traditional Medicine Clinic at Phra Pok Klao Hospital. The WCR comprises eight medicinal species: *Alpinia galanga* (L.) Willd., *Smilax glabra* Wall. ex Roxb., *Sm. corbularia* Kunth., *Smilax* sp., *Stemona involuta* Inthachub., *St. collinsae* Craib., *Rhinacanthus nasutus* (L.) Kurz., and *Acanthus ilicifolius* L. [1]. Previous observational data from Prapokklao Hospital suggested a high rate of clinical improvement among patients with psoriasis treated with the Wannachawee Recipe [1]. Although empirical results have been positive, the standardization process lacks sufficient scientific rigor.

The therapeutic efficacy of polyherbal recipes is inherently linked to the synergistic effects of complex secondary metabolites. Therefore, ensuring the authenticity and uniformity of raw materials, alongside rigorous control of the extraction and formulation processes, is critical for maintaining phytochemical consistency [2]. Comprehensive quality specifications are mandatory to facilitate reliable manufacturing, clinical traceability, and reproducibility [3,4,5]. While techniques such as High-Performance Liquid Chromatography (HPLC) fingerprinting and Thin-Layer Chromatography (TLC) are standard for monitoring herbal integrity, WCR presents a unique analytical challenge. In aqueous extracts, conventional TLC often yields poor visualization, necessitating the integration of more sensitive, multidimensional analytical platforms. In contemporary natural product chemistry, Compact Mass Spectrometry (CMS) has emerged as a robust tool for real-time, small-scale chemical analysis. Its capacity for rapid screening of complex mixtures and operational flexibility makes it an ideal accompaniment to traditional HPLC fingerprinting [6].

Although the WCR has been clinically applied in the treatment of psoriasis, scientific evidence supporting its chemical standardization and pharmacological activity remains limited. Identifying a reliable chemical marker and developing a validated analytical method for routine quality control are essential to ensure reproducibility and regulatory acceptance of polyherbal formulations. Moreover, linking chemical markers and biological activity is critical for demonstrating pharmacological relevance and substantiating clinical efficacy claims. This study aimed to establish comprehensive quality control parameters for the WCR by employing pharmacognostic authentication, physicochemical evaluation, and chromatographic fingerprinting. Additionally, it involved the development and validation of an analytical method to quantify *trans*-*p*-coumaryl alcohol as a key bioactive marker and the evaluation of its anti-inflammatory activity to support the pharmacological relevance of WCR.

## 2. Results

### 2.1. Macroscopic and Microscopic Identification

**Figure 1:**
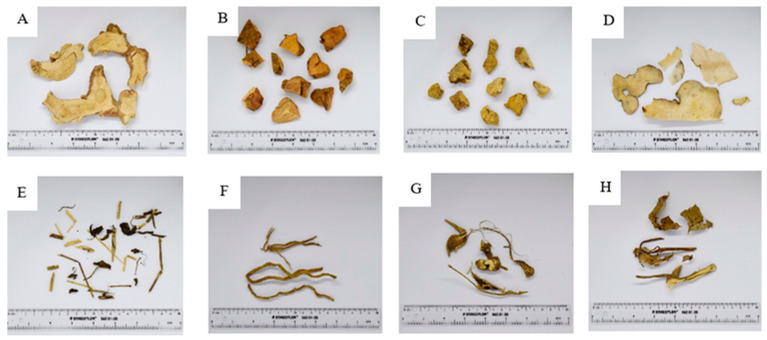
Macroscopic characteristics of crude drugs. (**A**) Rhizome of *Alpinia galanga* (L.) Wild. (**B**) Rhizome of *Smilax Corbularia* Kunth. (**C**) Rhizome of *Smilax glabra* Wall.ex Roxb. (**D**) Rhizome of *Smilax* sp. (**E**) Aerial part of *Rhinacanthus nasutus* (L.) Kurz. (**F**) Roots of *Stemona involuta* Inthachub.; (**G**) Roots of *Stemona collinsae* Craib. (**H**) Aerial part of *Acanthus ilicifolius* L.

The WCR comprises eight species. The macroscopic characteristics of these species are presented in Figure 1.

A. The rhizome of *Alpinia galanga* (L.) Wild. The underground rhizome is brown on the outside with distinct nodes. The flesh inside is white or slightly yellowish white with a distinctive smell.

B. The rhizome of *Smilax corbularia* Kunth. The underground rhizome is dry; the surface is not smooth; the outer surface is brown mixed with grey, with red flesh and a fine texture.

C. The rhizome of *Smilax glabra* Wall.ex Roxb. The underground rhizome is dry; the skin is not smooth; the outer skin is brown; the flesh is white and yellowish-brown, with a slightly sweet, salty taste.

D. The rhizome of *Smilax* sp. Thin dry sheets are smooth, white, with some areas that are light brown, fragile, and have a unique smell.

E. The aerial part of *Rhinacanthus nasutus* (L.) Kurz. Dry tree, branches will be brown-green, leaves are single leaves, oval, with a pointed leaf end, cone leaf, smooth leaf edge, dark brown leaf sheet.

F. The roots of *Stemona involuta* Inthachub. Dry roots, yellowish brown, white flesh, roots 10–20 cm long.

G. The roots of *Stemona collinsae* Craib. Dry root, yellowish brown, white inside, about 10 cm long, the center is more swollen than the head and tail, and has a distinctive smell.

H. The aerial part of *Acanthus ilicifolius* L. Dry, solid, brown on the outside, yellow-green on the inside. There are single-leaf, circular or parallel trunks. The edges of the leaves are thin and loose.

Microscopic analysis of WCR powder (Figure A9) revealed a complex array of diagnostic features from diverse plant tissues, including starch grains, parenchyma with crystals, trichomes, sclereids, and various vessel types. These anatomical structures represent the combined characteristics of the eight crude drugs included in the formulation. Table 1 provides a comparative summary that associates each microscopic element observed in Figure A9 with its likely herbal source among the WCR components. This correlation substantiates the presence and authenticity of all constituent herbs in the composite powder, thereby confirming the microscopic integrity of the formulation before chemical profiling.

**Figure A1:**
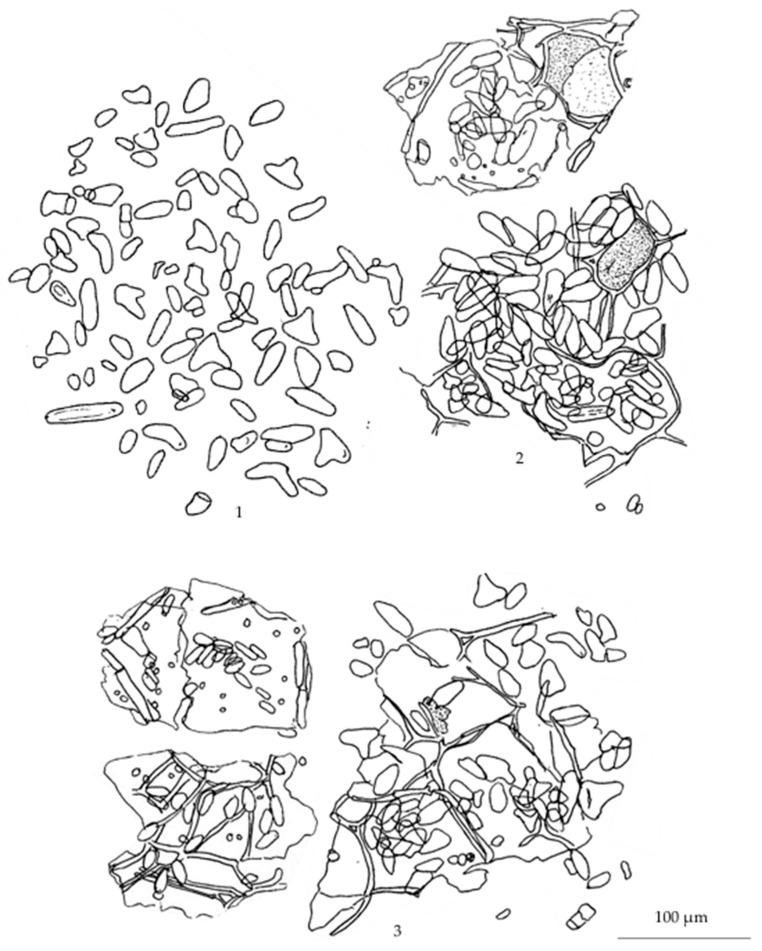

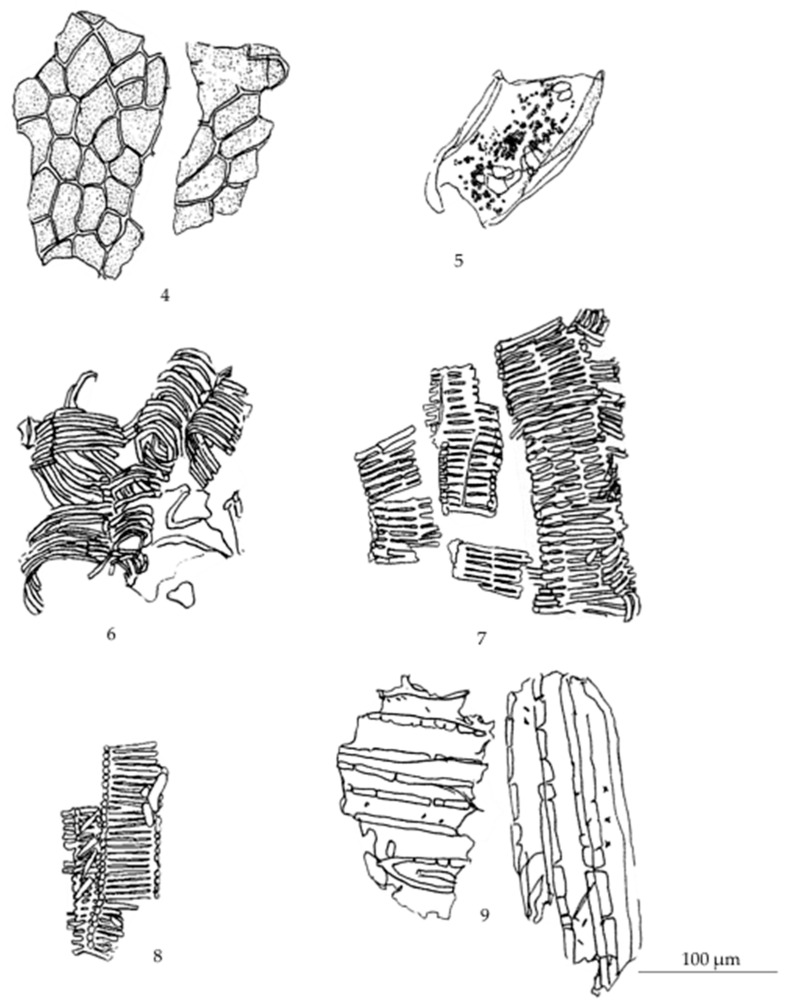
Diagnostic characteristics of *Alpinia galanga* (L.) Willd. rhizome (Kha) powder: (**1**) starch grains; (**2**) parenchyma containing starch grains and oil cell; (**3**) parenchyma containing starch grains; (**4**) epidermis; (**5**) parenchyma studded with silica crystalline matter; (**6**) spiral vessels; (**7**) scalariform vessels; (**8**) reticulated vessel and scalariform vessel; (**9**) fibers.

**Figure A2:**
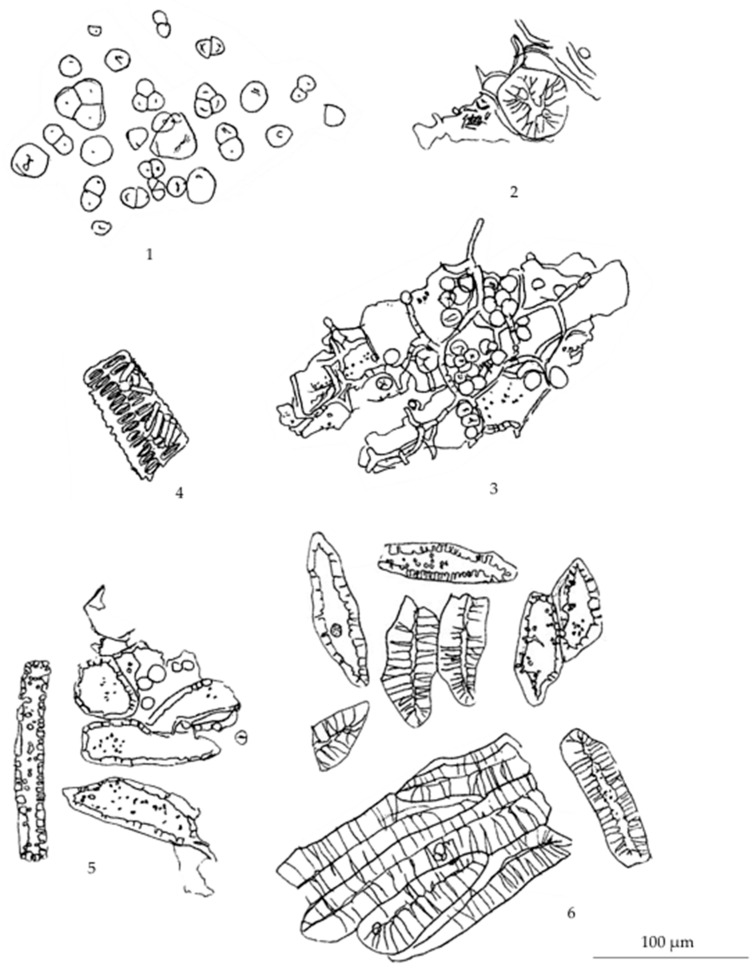
Diagnostic characteristics of *Smilax glabra* Wall.ex Roxb. powder: (**1**) starch grains; (**2**) stone cell and parenchyma containing rod crystals; (**3**) parenchyma containing starch grains; (**4**) bordered pitted vessel and parts of spiral vessel; (**5**) parenchyma; (**6**) sclereids.

**Figure A3:**
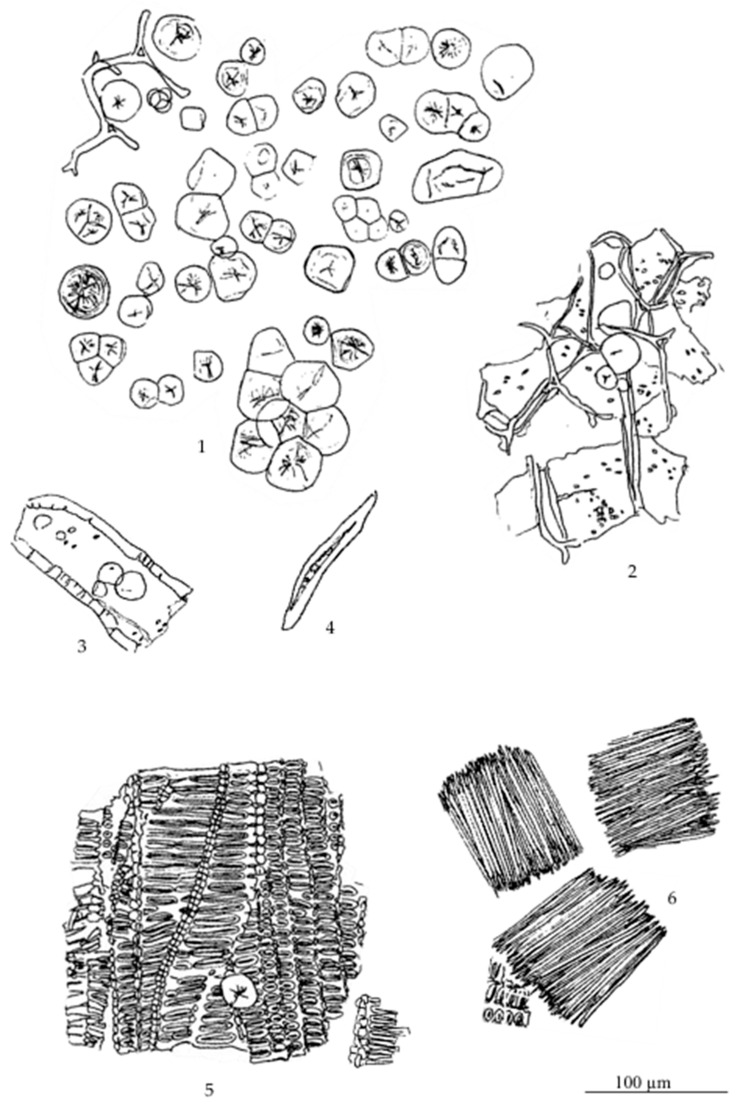
Diagnostic characteristics of *Smilax corbularia Kunth*. powder: (**1**) starch grains; (**2**) parenchyma; (**3**) macrosclereid; (**4**) fiber; (**5**) xylem elements showing reticulated vessels, bordered pitted vessels and scalariform vessels; (**6**) needle-shaped crystals.

**Figure A4:**
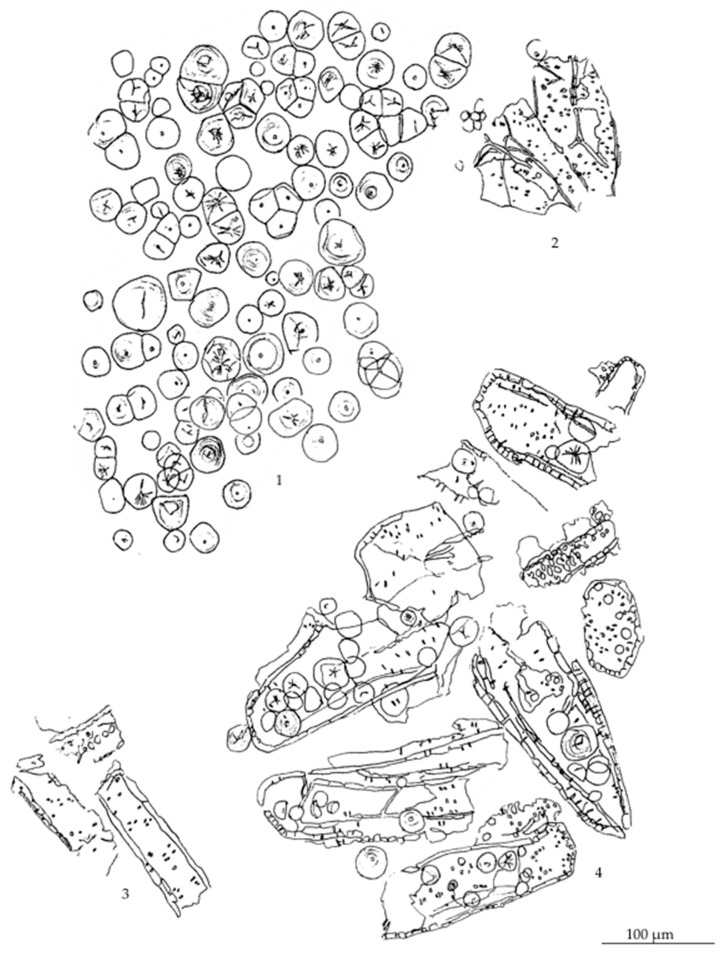

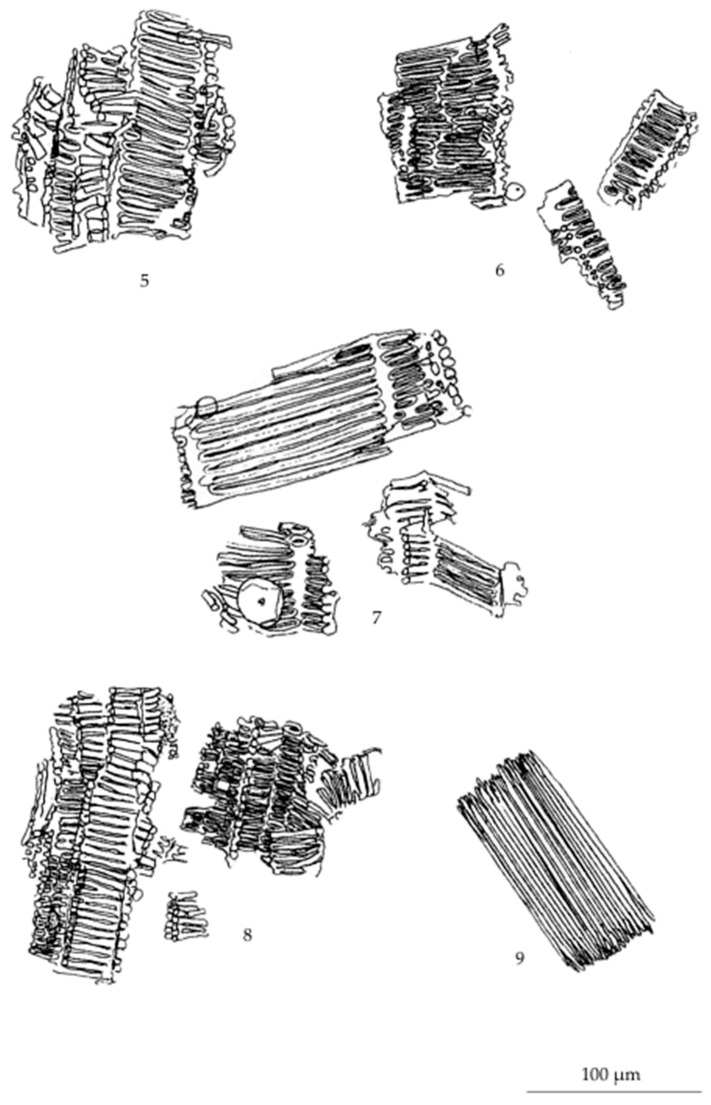
Diagnostic characteristics of *Smilax* sp. powder: (**1**) starch grains; (**2**) thin-walled parenchyma showing simple pits and intercellular spaces; (**3**) porous parenchyma; (**4**) macrosclereids containing starch grains showing intercellular spaces and simple pits; (**5**) xylem elements showing reticulated vessels and scalariform-bordered pitted vessels; (**6**) fragments of scalariform-bordered pitted vessels; (**7**) large scalariform-bordered pitted vessels; (**8**) bordered pitted vessels, reticulated vessels and scalariform vessels; (**9**) needle-shaped crystals.

**Figure A5:**
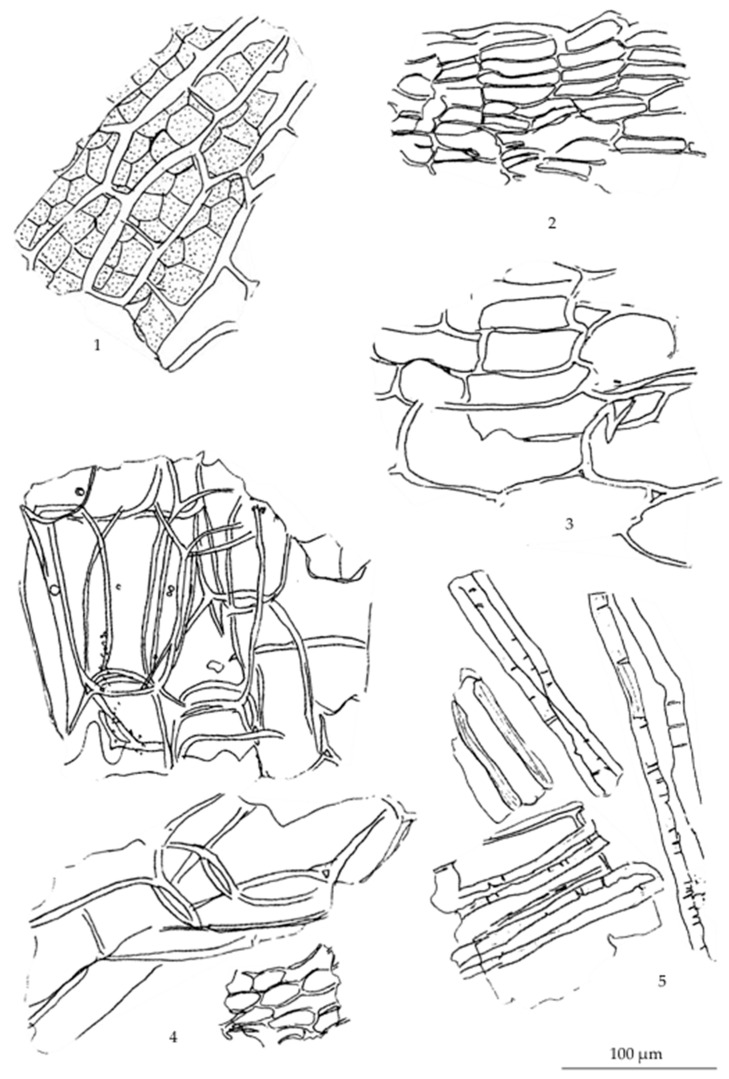

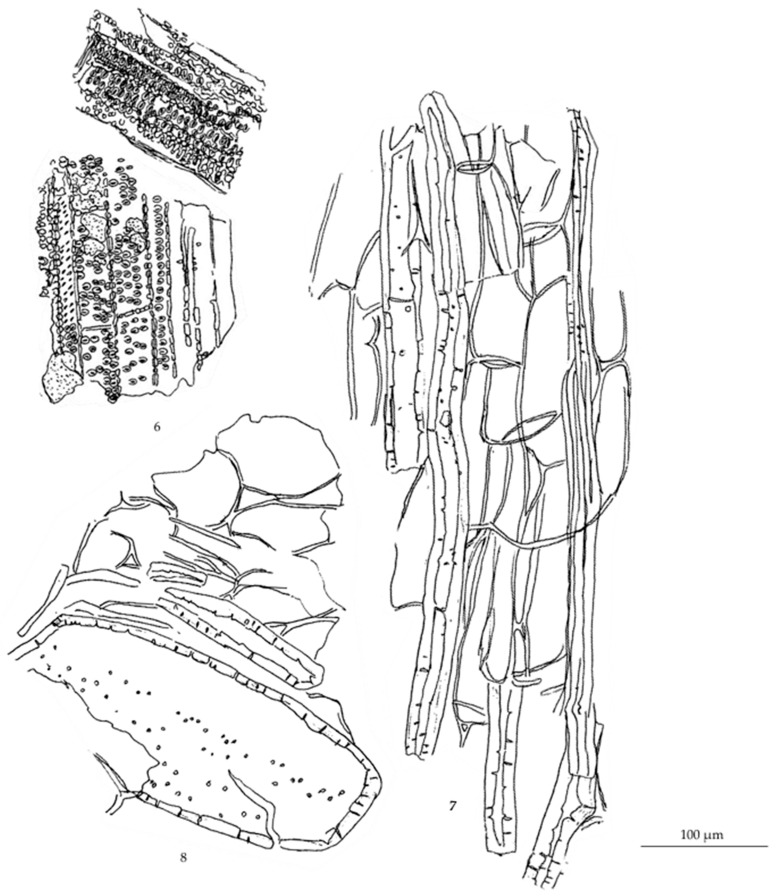
Diagnostic characteristics of *Stemona involuta* Inthachub. powder: (**1**) epidermis with under lying hypodermis containing greyish substances; (**2**) cork in sectional view; (**3**) phelloderm; (**4**) parenchyma; (**5**) fibers associated with parenchyma; (**6**) xylem elements showing tracheids, bordered pitted vessels and xylem parenchyma; (**7**) fibers associated with parenchyma; (**8**) macrosclereids and parenchyma.

**Figure A6:**
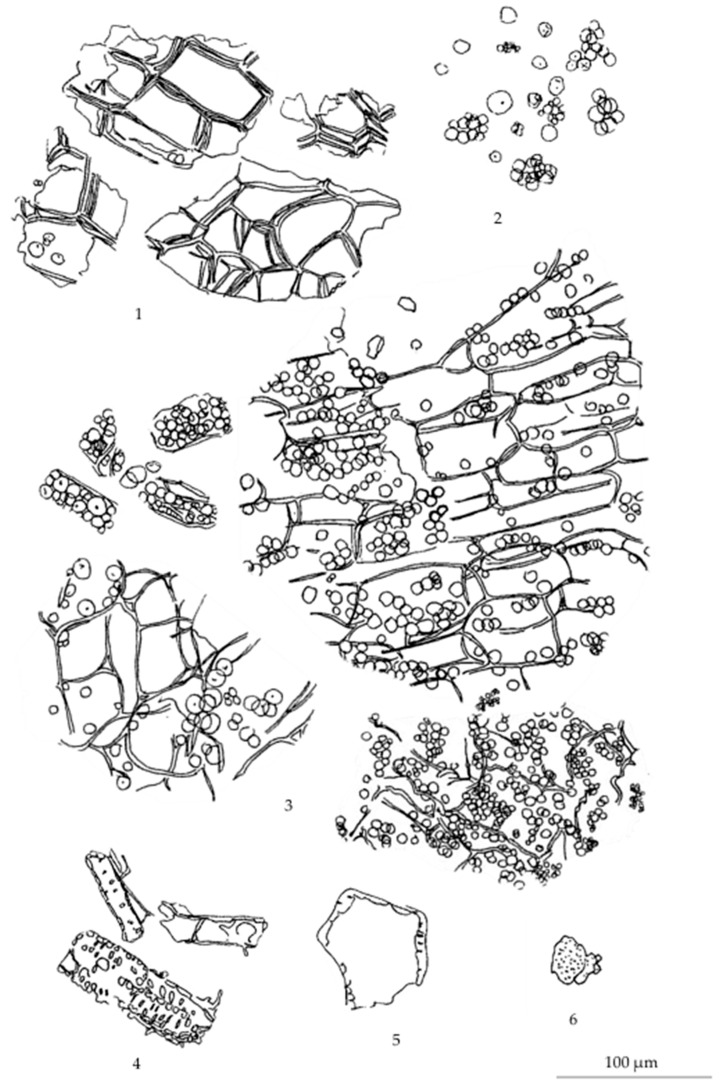

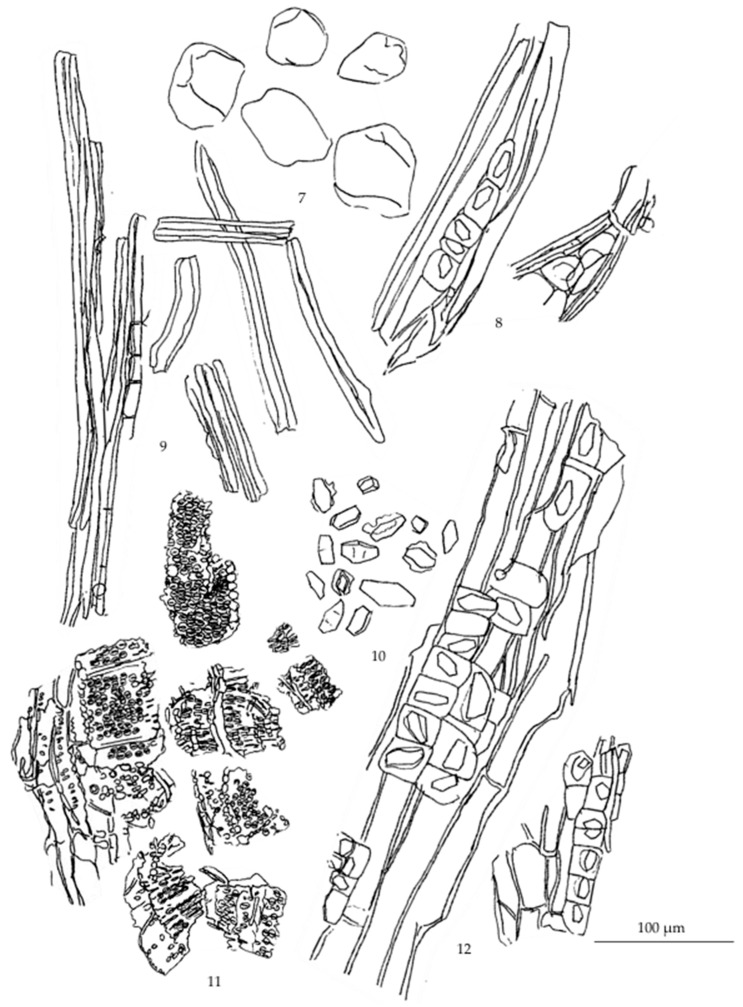
Diagnostic characteristics of *Stemona collinsae* Craib. powder: (**1**) corks; (**2**) starch grains; (**3**) parenchyma containing starch grains; (**4**) porous parenchyma; (**5**) macrosclereids; (**6**) greyish mass; (**7**) transparent masses; (**8**) bundle of fibers associated with parenchyma containing prismatic crystals; (**9**) fragments of fibers; (**10**) prismatic crystals; (**11**) bordered pitted vessels, some associated with porous parenchyma; (**12**) parenchyma containing prismatic crystals associated with thin-wall parenchyma.

**Figure A7:**
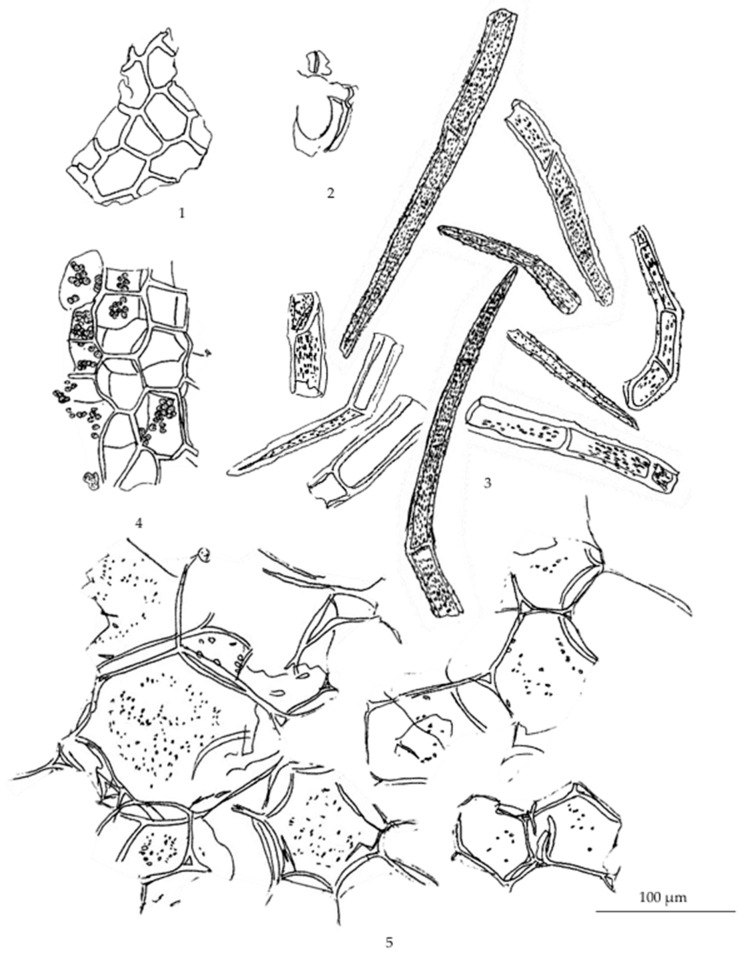

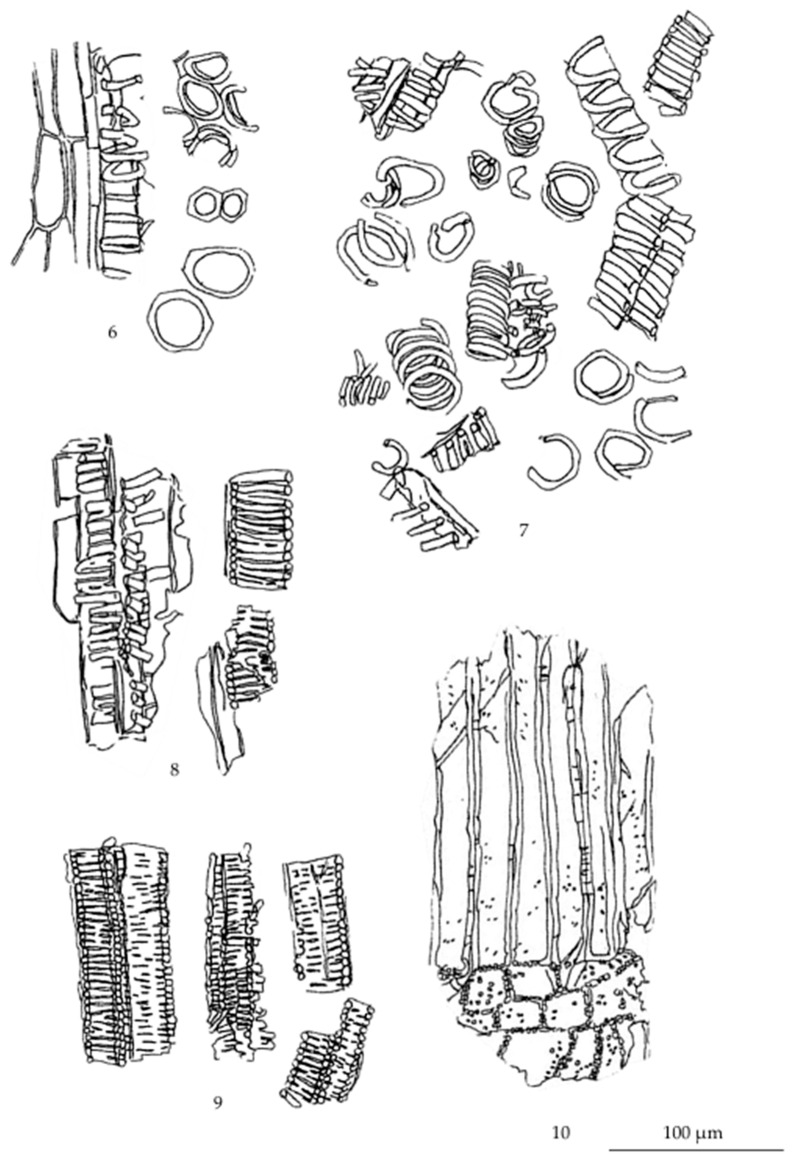
Diagnostic characteristics of *Rhinacanthus nasutus* (L.) Kurz. powder: (**1**) epidermis; (**2**) diacytic stoma; (**3**) trichome; (**4**) chlorenchyma; (**5**) pith parenchyma; (**6**) annular vessels; (**7**) spiral vessels; (**8**) reticulated vessels; (**9**) pitted vessels associated with reticulated vessels; (**10**) medullary ray with underlying sclereids.

**Figure A8:**
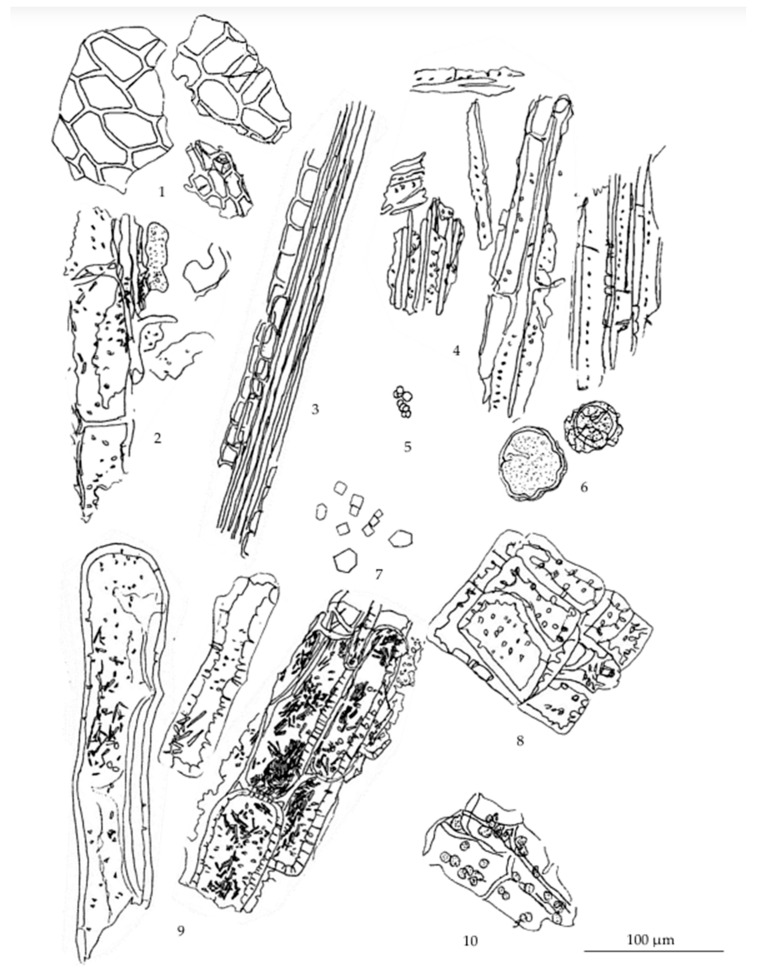

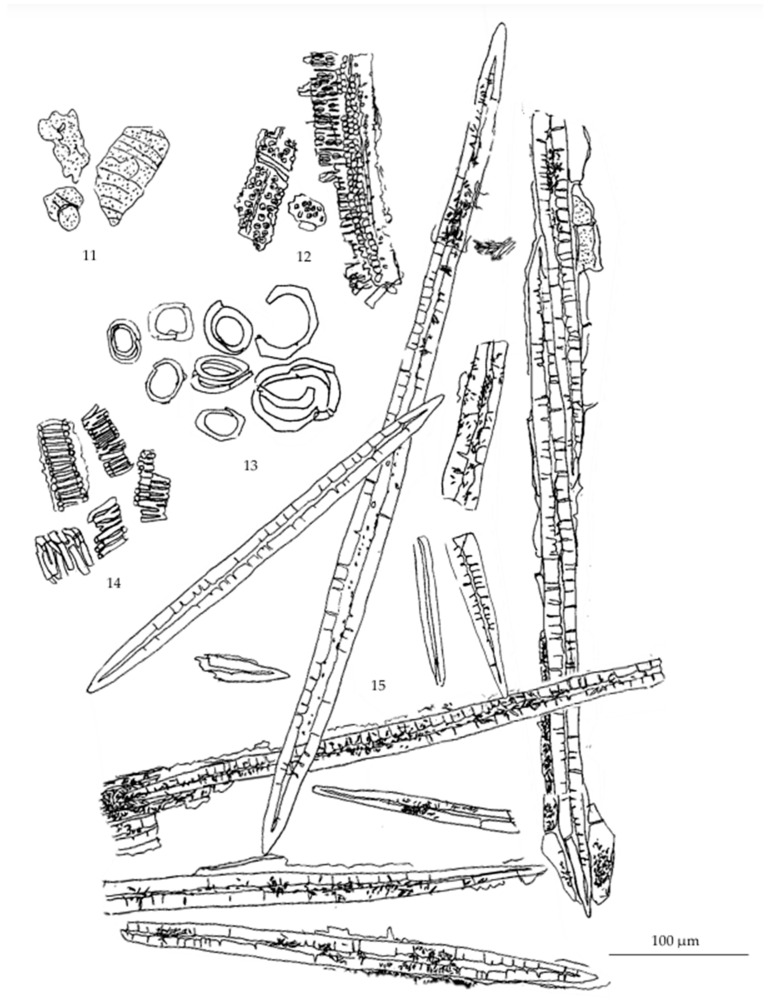
Diagnostic characteristics of *Acanthus ilicifolius* L. powder: (**1**) epidermis; (**2**) parenchyma; (**3**) collenchyma associated with epidermis in longitudinal view; (**4**) collenchyma; (**5**) starch grains; (**6**) cystolith; (**7**) prismatic crystals; (**8**) porous parenchyma; (**9**) macrosclereid containing rod crystals; (**10**) chlorenchyma; (**11**) reddish brown substances; (**12**) bordered pitted vessels; (**13**) parts of spiral vessels; (**14**) fragments of reticulated vessels; (**15**) fibers, with some associated with parenchyma containing rod crystals.

Microscopic examination of the Wannachawee recipe confirmed the presence of all eight medicinal plant species (see Figure A1, Figure A2, Figure A3, Figure A4, Figure A5, Figure A6, Figure A7 and Figure A8). Distinct microscopic characteristics were identified for each species. For instance, the size of starch grains serves as an indicator of plant type. Small starch grains, often associated with parenchyma, were observed in *S. collinsae* (Non Tai Yak). In contrast, *A. galanga* (Kha) exhibited long, oval-shaped starch grains. These diagnostic features are summarized in Table 2.

### 2.2. Physical and Chemical Identification

The quality assessment of the WCR included measurements of ethanol and water extract contents, moisture content, total ash, and acid-insoluble ash. These parameters represent the physicochemical properties of the formulation and were evaluated in accordance with the Thai Herbal Pharmacopoeia (2018) standards [7], as presented in Table 3.

### 2.3. Compact Mass Spectrometry

The compounds identified in the WCR extract were analyzed using compact mass spectrometry (CMS) in Selected Ion Monitoring (SIM) mode with atmospheric pressure chemical ionization (APCI) in positive ionization. Table 4 presents each compound’s molecular mass, ionized mass [M+H]+, relative intensity, and corresponding herbal source within the recipe. These findings confirm the presence of multiple constituents that contribute to the therapeutic profile of WCR.

**Figure 2:**
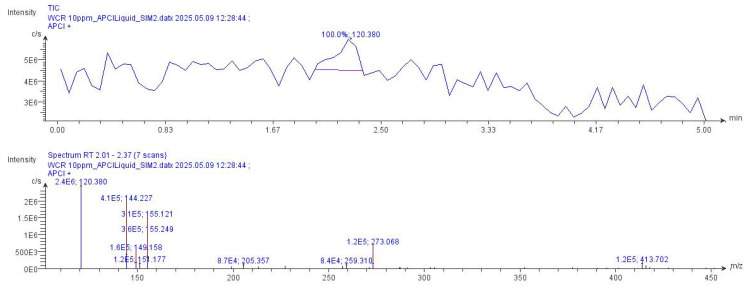
Chromatogram and spectrum of the active compounds [M+H]^+^ found in the WCR extract.

Representative chromatograms and mass spectra of the active compounds detected in the WCR extract by CMS are presented in Figure 2. The observed peaks correspond to the [M+H]+ ions of key constituents, further confirming their identification and abundance in the recipe.

### 2.4. Chromatographic Fingerprint by HPLC

**Figure 3:**
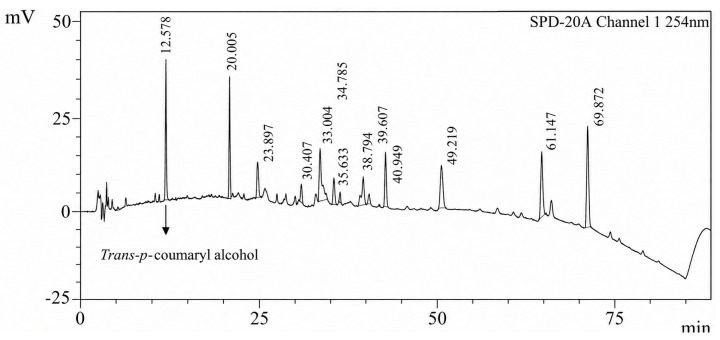
Chromatogram of the WCR extract of 1 mg/mL concentration at a wavelength of 254 nm.

The chromatographic fingerprint of the WCR extract, obtained by High-Performance Liquid Chromatography (HPLC) at a concentration of 1 mg/mL and a detection wavelength of 254 nm, provides critical insights into its chemical composition. The chromatogram (Figure 3) displays multiple peaks, each corresponding to distinct phytochemicals present in the WCR extract. The main peaks appear at retention times of 12.578 and 20.005, suggesting the presence of major compounds in WCR. This fingerprint profile serves as a chemical signature for WCR, facilitating quality control and ensuring batch-to-batch consistency.

### 2.5. HPLC Method Validation Results 

#### 2.5.1. Specificity

The specificity of the developed HPLC method was assessed to confirm accurate identification of the analyte despite the presence of matrix components and potential interfering substances. Representative chromatograms obtained under optimized conditions are shown in Figure 3.

**Figure 4:**
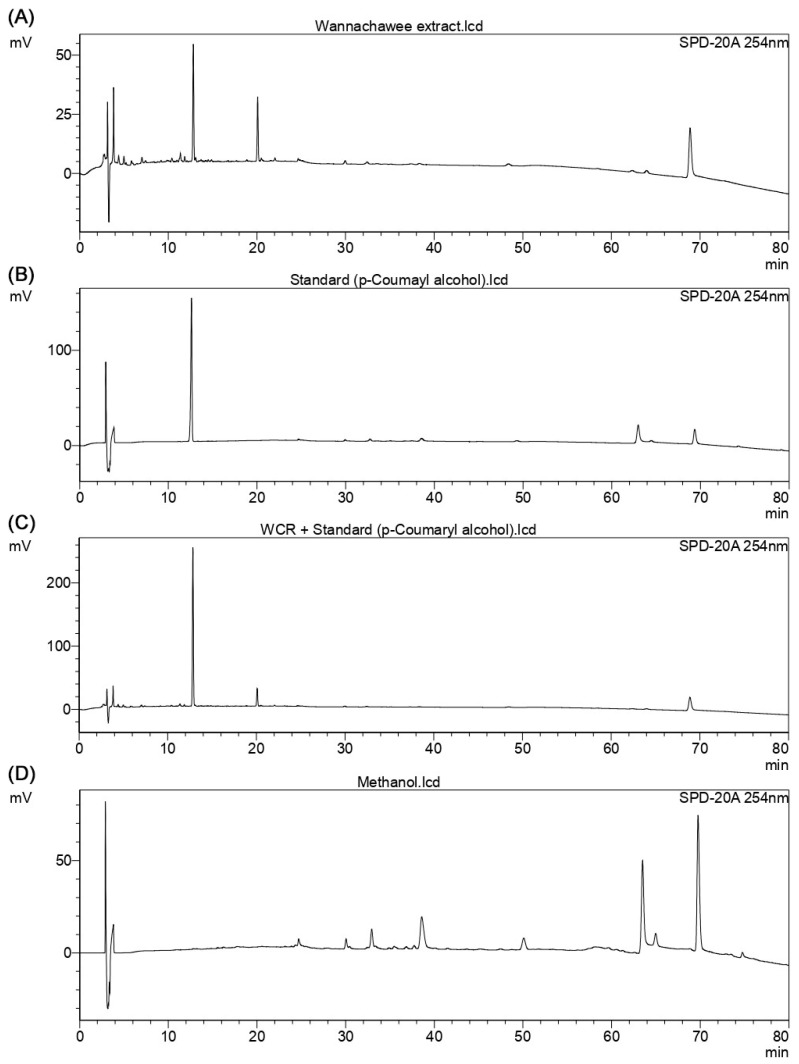
Chromatograms obtained for the evaluation of method specificity: (**A**) sample extract, (**B**) standard solution of *trans*-*p*-coumaryl alcohol, (**C**) sample extract spiked with *trans*-*p*-coumaryl alcohol, and (**D**) methanol (blank), recorded at 254 nm under the optimized HPLC conditions.

The specificity of the proposed HPLC method was evaluated by comparing chromatograms of methanol (blank), the standard solution of *trans*-*p*-coumaryl alcohol, the sample extract, and the sample extract spiked with the standard under optimized chromatographic conditions with detection performed at 254 nm (Figure 4).

The results confirm that the method is specific and can unequivocally assess *trans*-*p*-coumaryl alcohol in the presence of matrix components.

#### 2.5.2. Linearity

**Figure 5:**
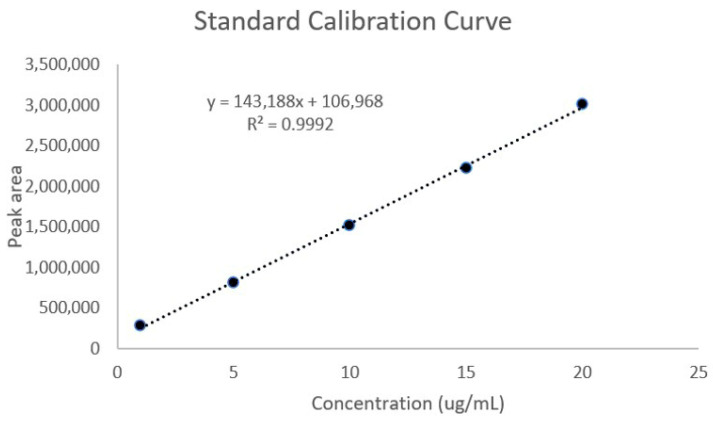
Standard calibration curve of *trans*-*p*-coumaryl alcohol showing the linear relationship between peak area and concentration in the range of 1–20 µg/mL. The regression equation was *y* = 143,188*x* + 106,968, with a correlation coefficient (R^2^) of 0.9992. Data represent mean peak areas obtained from triplicate injections.

A standard calibration curve of *trans*-*p*-coumaryl alcohol was constructed over the concentration range of 1–20 µg/mL to assess the relationship between peak area and analyte concentration (Figure 5).

The calibration curve of *trans*-*p*-coumaryl alcohol exhibited a linear relationship between peak area and concentration over the range of 1–20 µg/mL, as shown in Figure 5. The linear regression equation was *y* = 143,188*x* + 106,968, with a correlation coefficient (R^2^) of 0.9992.

#### 2.5.3. LOD and LOQ

The limits of detection (LOD) and quantification (LOQ) of *trans*-*p*-coumaryl alcohol were determined based on the standard deviation of the intercept and the slope of the calibration curve. The LOD and LOQ were 0.64 µg/mL and 1.94 µg/mL, respectively.

#### 2.5.4. Accuracy

Method accuracy was determined by recovery experiments at three concentration levels (5, 10, and 20 µg/mL), each analyzed in triplicate. The percentage recovery was calculated by comparing the measured concentration with the nominal value, and the precision of recovery was expressed as %RSD. The obtained results are presented in Table 5.

The accuracy of the developed method was evaluated by recovery studies at three concentration levels (low, medium, and high), each analyzed in triplicate. As shown in Table 5, the percentage recoveries of *trans*-*p*-coumaryl alcohol ranged from 101.30% to 108.60%, with %RSD values between 0.87% and 1.46%.

#### 2.5.5. Precision

The precision of the method was evaluated in terms of repeatability (intraday) and intermediate precision (interday). Intraday precision was assessed by analyzing three concentration levels (5, 10, and 20 µg/mL), each in five replicates within a single day. Interday precision was evaluated over three consecutive days, with three replicates at each concentration level per day. The results are presented in Table 6 and Table 7.

The intraday precision of the method was evaluated by analyzing three concentration levels (5, 10, and 20 µg/mL), each in five replicates within a single day. As shown in Table 6, the mean measured concentrations were 4.83 ± 0.01, 9.48 ± 0.04, and 20.89 ± 0.05 µg/mL at 5, 10, and 20 µg/mL, respectively. The %RSD values were 0.11%, 0.46%, and 0.22%, respectively.

The interday precision of the method was evaluated over three consecutive days at three concentration levels (5, 10, and 20 µg/mL), with three replicates per concentration per day. As shown in Table 7, the mean measured concentrations for Day 1 ranged from 5.36 ± 0.01 to 21.27 ± 0.01 µg/mL, with %RSD values between 0.05% and 0.23%. For Day 2, the mean concentrations ranged from 4.93 ± 0.01 to 20.99 ± 0.02 µg/mL, with %RSD values between 0.10% and 0.25%. For Day 3, the mean concentrations ranged from 4.89 ± 0.01 to 20.63 ± 0.02 µg/mL, with %RSD values between 0.11% and 0.31%.

### 2.6. Nitric Oxide Inhibitory Activity

#### 2.6.1. Cell Viability of RAW 264.7 Macrophages

**Figure 6:**
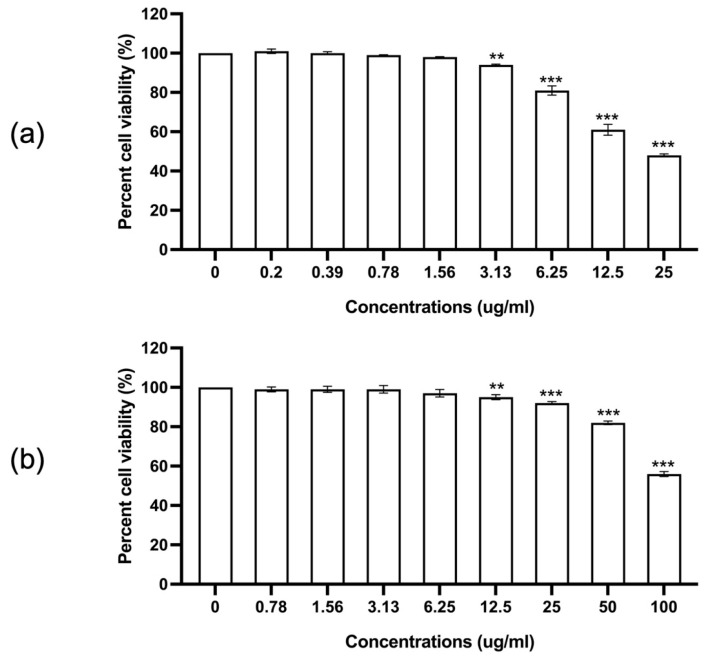
Effects of *trans*-*p*-coumaryl alcohol (**a**) and indomethacin (**b**) on the proliferation of RAW 264.7 cells at 24 h. Data are presented as mean ± SEM from three independent experiments. * indicates a statistically significant difference compared with the control group (0 concentration); ** *p* < 0.01, and *** *p* < 0.001.

The cytotoxicity of *trans*-*p*-coumaryl alcohol and indomethacin on RAW 264.7 macrophages was evaluated using a cell proliferation assay after 24 h of incubation. As shown in Figure 6, *trans*-*p*-coumaryl alcohol exhibited a concentration-dependent reduction in cell viability. Significant cytotoxicity was observed at concentrations ≥ 3.13 µg/mL; however, cell viability remained above 80% at concentrations ≤ 6.25 µg/mL.

Indomethacin also showed a concentration-dependent effect on cell viability, with significant cytotoxicity observed at concentrations ≥ 12.5 µg/mL, while concentrations ≤ 50 µg/mL maintained cell viability above 80%.

Based on these results, concentrations that maintained cell viability above 80% were selected for subsequent nitric oxide inhibition assays to ensure that the observed effects were not attributable to cytotoxicity.

#### 2.6.2. Inhibitory Effect on Nitric Oxide Production

**Figure 7:**
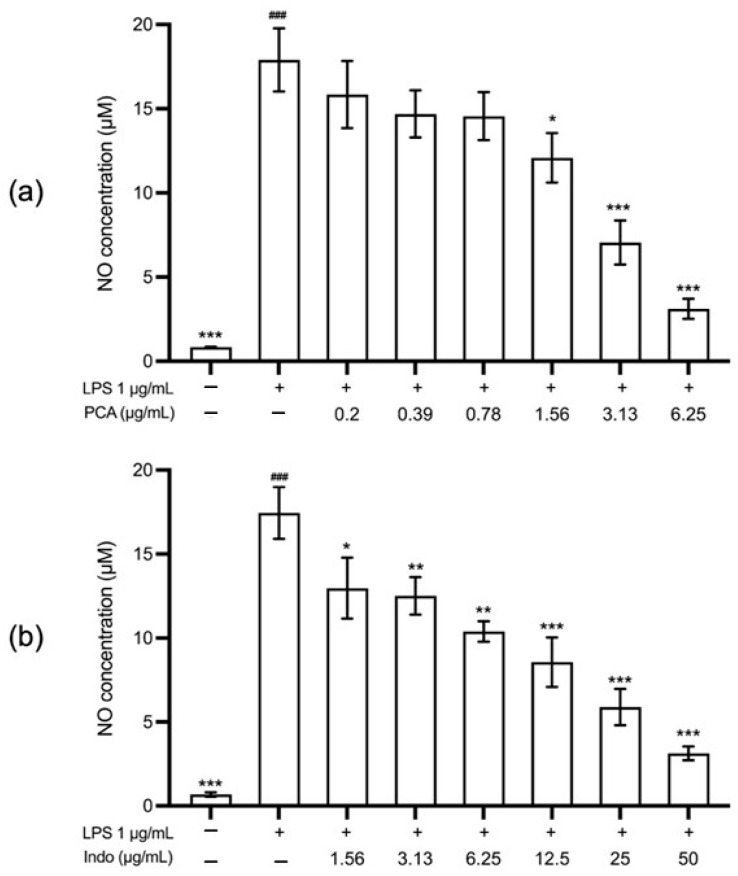
Effects of *trans*-*p*-coumaryl alcohol (**a**) and indomethacin (**b**) on anti-inflammatory activity at 24 h. The anti-inflammatory effect is directly proportional to the percentage reduction in nitric oxide (NO) level. Data are presented as mean ± SEM from three independent experiments. # indicates a statistically significant difference between LPS-stimulated cells and the non-stimulated control cells; ### *p* < 0.001. * indicates a statistically significant difference compared with LPS-stimulated cells; * *p* < 0.05, ** *p* < 0.01, *** *p* < 0.001.

Lipopolysaccharide (LPS) stimulation significantly increased nitric oxide (NO) production in RAW 264.7 macrophages compared with unstimulated control cells (*p* < 0.001). Following treatment with *trans*-*p*-coumaryl alcohol, NO production was significantly reduced at all tested concentrations and in a concentration-dependent manner (Figure 7).

At the highest non-cytotoxic concentration (6.25 µg/mL), *trans*-*p*-coumaryl alcohol reduced NO production to 17.16 ± 1.46% of the LPS-induced control. This inhibitory effect was comparable to that of indomethacin at 50 µg/mL, which reduced NO production to 17.79 ± 0.71%.

#### 2.6.3. Comparison of NO Inhibitory Activity with Indomethacin

**Figure 8:**
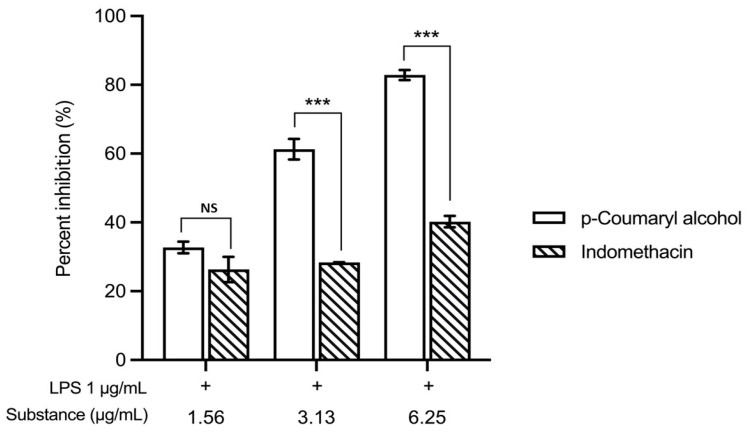
Effects of *trans*-*p*-coumaryl alcohol and indomethacin on nitric oxide (NO) production in LPS-stimulated RAW 264.7 macrophages treated with concentrations of 1.56–6.25 µg/mL in the presence of LPS (1 µg/mL) for 24 h. Data are presented as mean ± SEM (*n* = 3). Statistical analysis was performed using two-way ANOVA followed by Tukey’s post hoc test. *** *p* < 0.001 compared with indomethacin at the same concentration. NS, not significant.

When comparing the percentage inhibition of nitric oxide production at equivalent concentrations, *trans*-*p*-coumaryl alcohol demonstrated greater inhibitory activity than indomethacin within the concentration range of 1.56–6.25 µg/mL (Figure 8).

Specifically, *trans*-*p*-coumaryl alcohol exhibited NO inhibition rates ranging from 32.69% to 82.84%, whereas indomethacin showed inhibition rates ranging from 26.29% to 40.19% at corresponding concentrations. *Trans*-*p*-coumaryl alcohol exhibited significantly higher NO inhibitory activity than indomethacin at 3.13 and 6.25 µg/mL (*** *p* < 0.001), whereas no significant difference was observed at 1.56 µg/mL.

### 2.7. Quantification of trans-p-Coumaryl Alcohol

The validated HPLC method was successfully applied to determine the *trans*-*p*-coumaryl alcohol content in the Wannachawee formulation. The amount of *trans*-*p*-coumaryl alcohol in the extract was 8.77 ± 0.14 mg/g extract (mean ± S.D., *n* = 3).

## 3. Discussion

One of the primary challenges in developing the Wannachawee Recipe (WCR) as a scientifically validated medicine is its inherent complexity as a multi-herbal formulation. Each of the eight constituent crude drugs contains numerous secondary metabolites, whose composition can vary with factors such as plant authenticity, geographic origin, harvest season, storage conditions, and processing methods. This variability complicates efforts to achieve consistent quality, reproducibility, and efficacy [8]. Furthermore, there is a risk of adulteration, contamination, or substitution with morphologically similar but pharmacologically distinct materials, which may compromise both safety and therapeutic value [9]. Traditional preparations, such as WCR, frequently lack standardized chemical markers and quantitative quality parameters, making it challenging to monitor batch-to-batch uniformity in clinical or commercial production [10]. Consequently, establishing a clear specification is essential. A well-defined specification provides measurable criteria, including macroscopic, microscopic, physicochemical, and phytochemical markers, to ensure the authenticity and integrity of the herbal ingredients. Such specifications also support reproducibility in research and industry, and facilitate compliance with regulatory requirements outlined in the Thai Herbal Pharmacopoeia and World Health Organization guidelines. By defining parameters such as extractive values, moisture and ash contents, and chemical fingerprints, we can minimize variability, enhance consumer confidence, and strengthen the credibility of WCR as a standardized traditional medicine with broader application potential.

**Figure A9:**
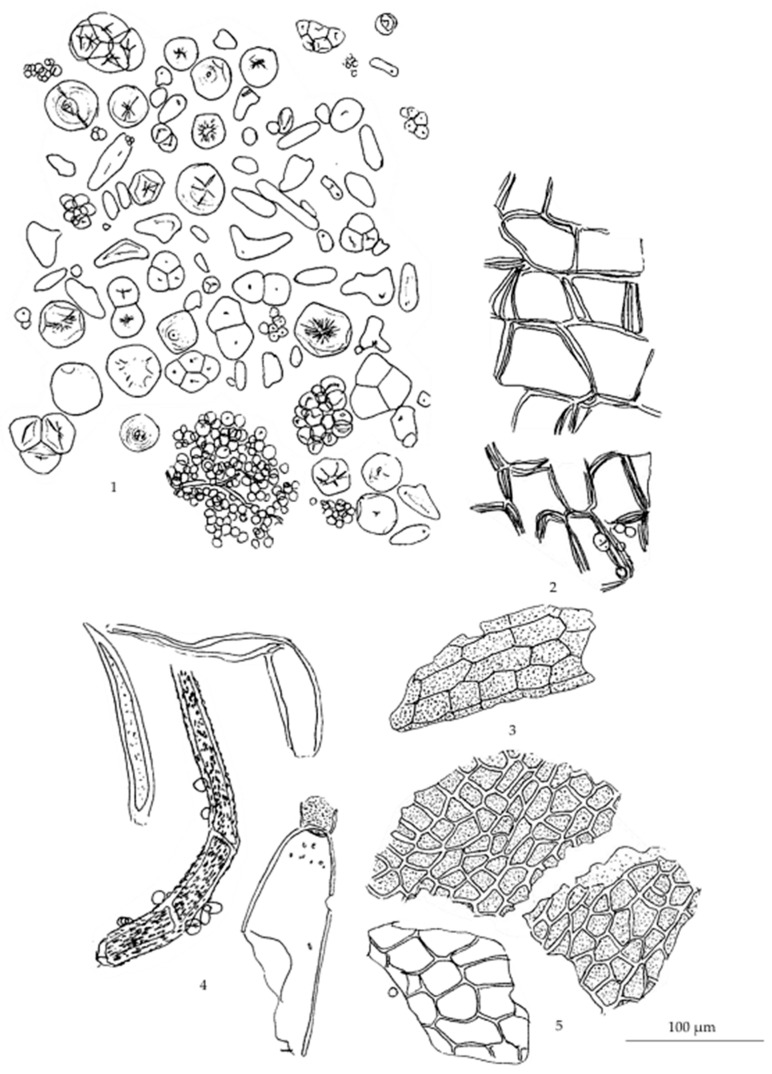

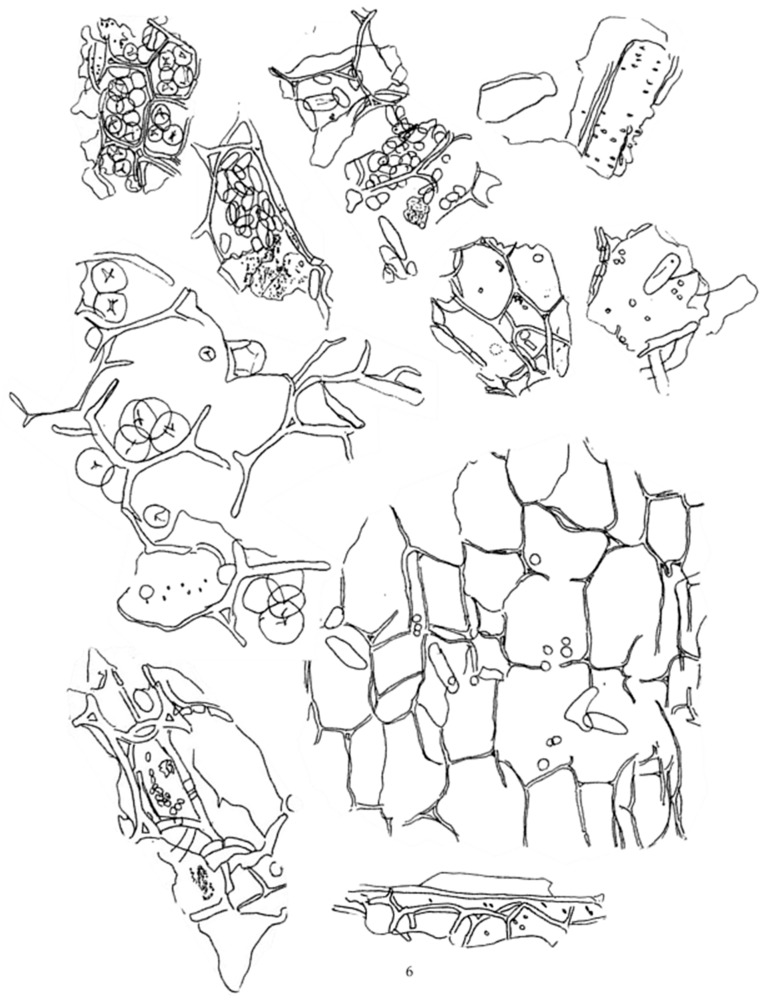

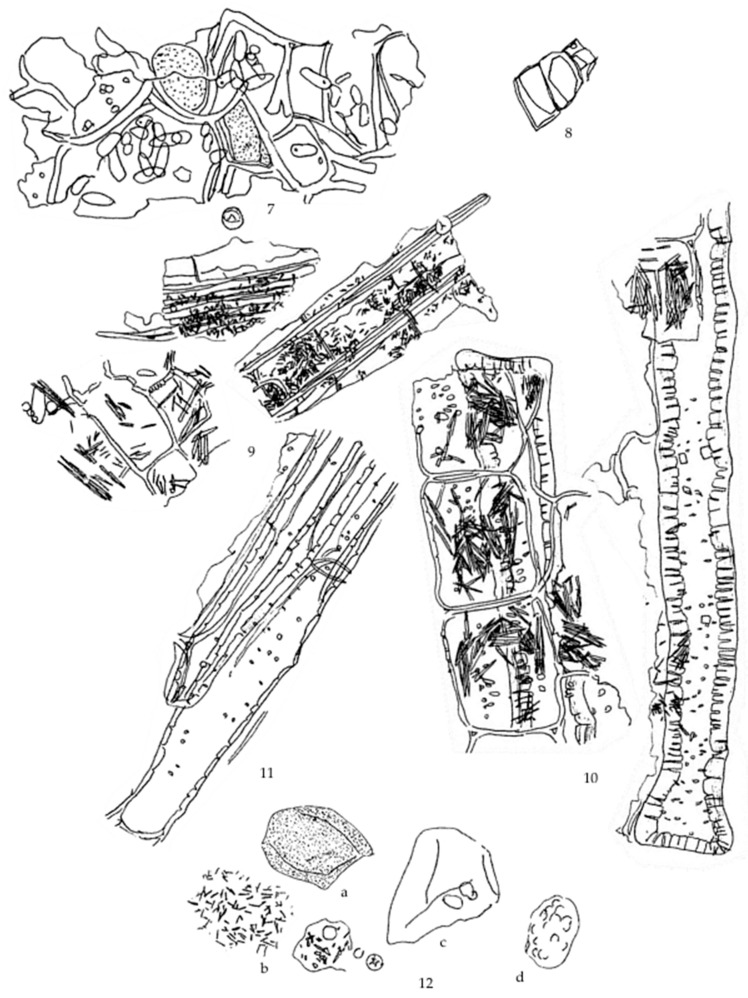

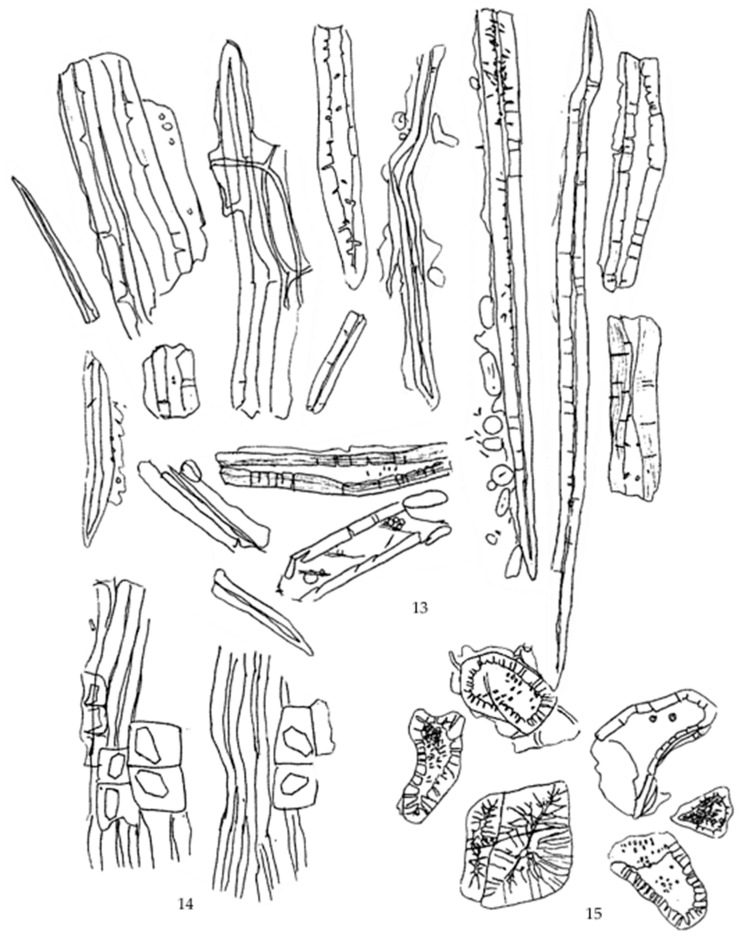

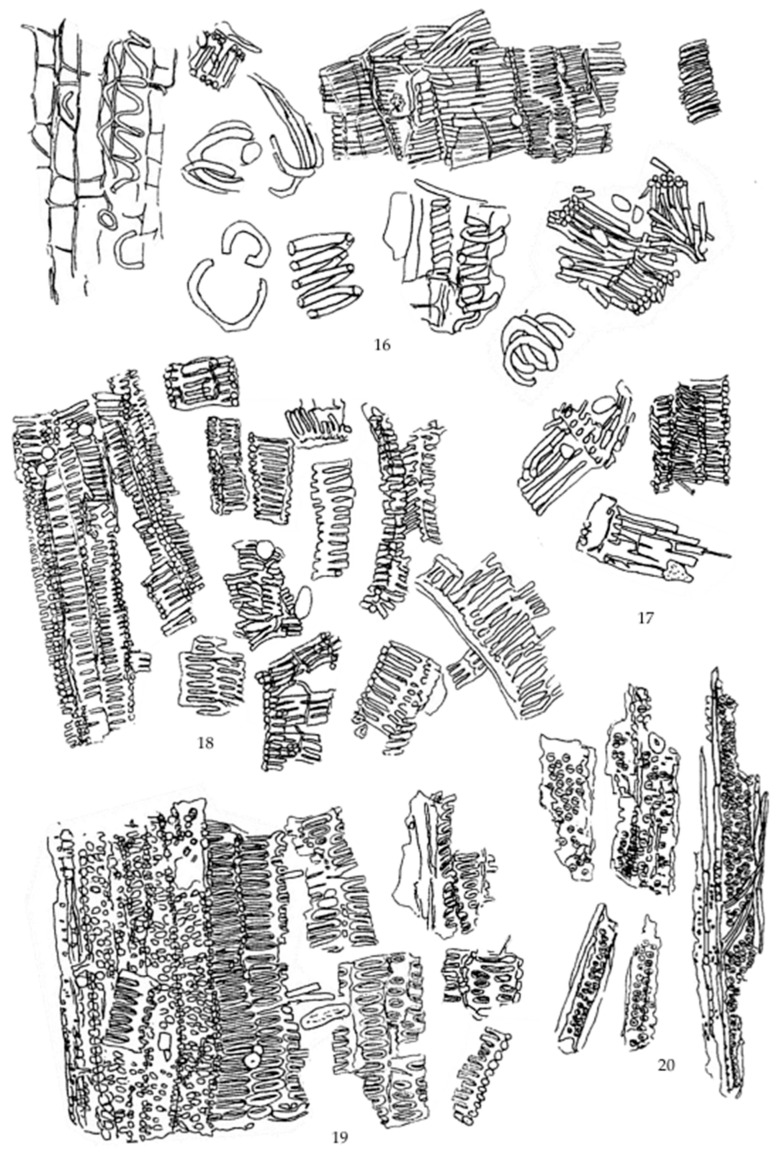
Diagnostic characteristics of Wannachawee recipe powder: (**1**) starch grains; (**2**) cork; (**3**) hypodermis containing greyish substances; (**4**) trichome; (**5**) epidermis; (**6**) parenchyma containing starch grains; (**7**) parenchyma containing starch grains and oil cells; (**8**) parenchyma containing prismatic crystals; (**9**) parenchyma containing rod crystals attached to fibers; (**10**) parenchyma containing needle shape crystals attach to large elongated macrosclereid; (**11**) elongated macrosclereid; (**12**) cell accumulants (a. reddish brown substance; b. rod crystals; c. transparent matter; d. cystolith); (**13**) fragments of fibers; (**14**) bundle of fibers and prism sheath; (**15**) sclereids, with some containing rod crystals; (**16**) spiral vessels; (**17**) reticulated vessels; (**18**) scalariform vessels; (**19**) scalariform-bordered pitted vessels, with some associated with porous parenchyma; (**20**) border-pitted vessels.

**Figure A10:**
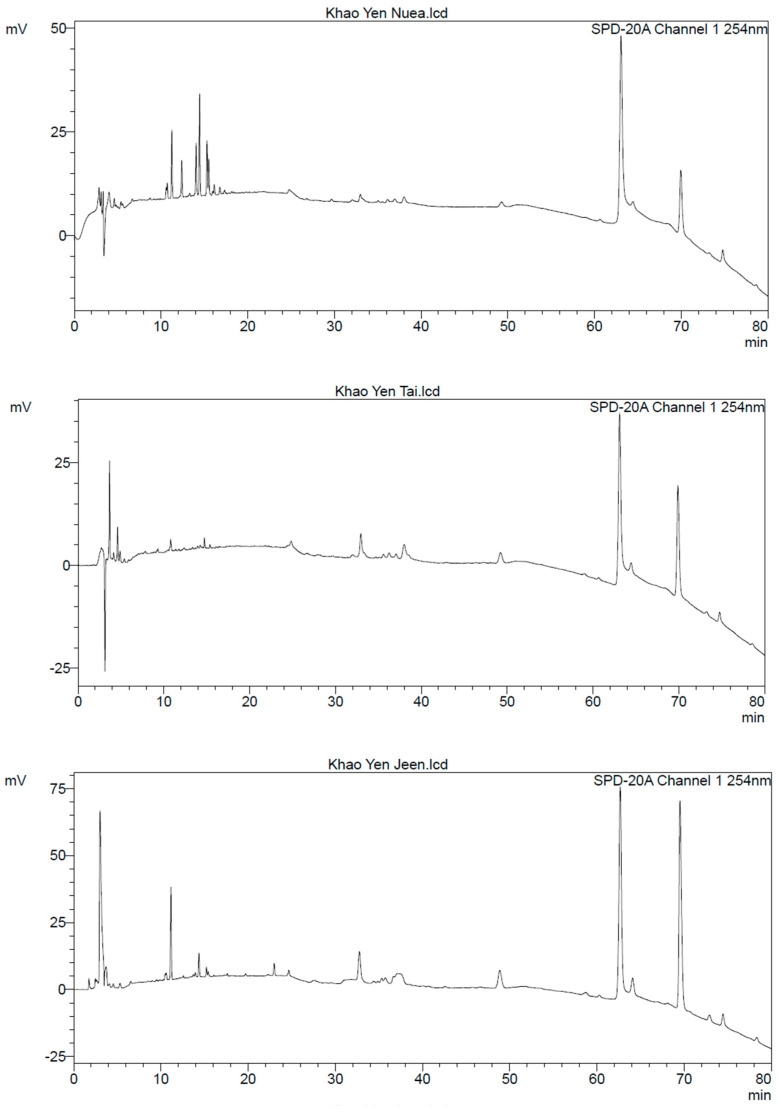
Chromatographic profiles of Khao Yen Nuea (*Smilax corbularia* Kunth.), Khao Yen Tai (*Smilax glabra* Wall.ex Roxb.), and Khao Yen Jeen (*Smilax* sp.) extracts analyzed by HPLC at a detector wavelength of 254 nm.

Macroscopic and microscopic examinations provided critical evidence to authenticate and ensure the quality control of crude drugs used in the Wannachawee Recipe (WCR). As illustrated in Figure 1, macroscopic evaluation identified distinct morphological characteristics for each herbal component, including the nodulated rhizome and aromatic odor of *Alpinia galanga*, the reddish flesh of *Smilax corbularia*, and the swollen roots of *Stemona collinsae*. These physical traits serve as primary diagnostic markers for distinguishing the eight species included in WCR, enabling reliable identification of each crude drug prior to further processing. Microscopic analysis of WCR powder (Figure A9) revealed diagnostic structures, including starch grains, trichomes, sclereids, and various vessel types, indicating the coexistence of tissues from all eight crude drugs. As summarized in Table 1, these anatomical features directly correlate with those of the individual herbal ingredients, confirming both authenticity and homogeneity of the formulation. Table 2 further details representative microscopic characteristics for each crude drug, illustrating diagnostic features specific to each herbal component in WCR. Each species exhibited characteristic elements, including starch grains, sclereids, vessels, trichomes, and cystoliths, which were confirmed by a botanist from the Faculty of Pharmacy at Chiang Mai University. These findings demonstrate that microscopic profiles effectively support species identification, purity assessment, and overall quality control of the WCR formulation. Among the eight herbal components, the *Smilax* group (Khao Yen Nuea, Khao Yen Tai, and Khao Yen Jeen) posed the greatest challenge in microscopic and taxonomic identification. In the Thai herbal market, considerable confusion exists regarding Khao Yen Nuea and Khao Yen Tai, as crude drugs sold under these names are most commonly derived from three genera: *Smilax*, *Dioscorea*, and *Pygmaeopremna* [11]. Identification of *Smilax* species in WCR was complicated by overlapping morphological and microscopic characteristics. Macroscopic observation (Figure 1) showed that the rhizome of *Smilax* sp. (Figure 1D) was lighter in color compared to those of *S. corbularia* and *S. glabra*, potentially due to differences in drying conditions, plant maturity, or post-harvest processing. Microscopic analysis revealed that starch grains of *Smilax* sp. (Figure A4) were large and comparable in size to those of *S. corbularia* (Figure A3), but larger than those in *S. glabra* (Figure A2). Additionally, the needle-shaped crystals (raphides) of *Smilax* sp. (Figure A4, image 9) were larger than those in *S. corbularia*, while such crystals were absent in *S. glabra*. The hilum of starch grains in *S. glabra* appeared as a distinct dot, contrasting with the stellate hilum observed in *Smilax* sp. and *S. corbularia*. HPLC chromatograms from the three *Smilax* species (Figure A10) indicated that the chemical profile of *Smilax* sp. more closely resembled that of *S. glabra* (Khao Yen Tai) than *S. corbularia* (Khao Yen Nuea). These findings underscore the taxonomic complexity within the *Smilax* group and highlight the necessity of integrated morphological and chemical approaches for accurate authentication of Khao Yen species in WCR. Microscopic observations not only validate the inclusion of the prescribed plant species but also provide an effective means of detecting adulteration or substitution, which are prevalent issues in traditional medicine. Collectively, macroscopic and microscopic evaluations demonstrate the robustness of combining morphological and anatomical markers as a dual approach to raw material authentication. This integrated examination ensures botanical accuracy of WCR ingredients, which is essential for maintaining reproducibility, safety, and therapeutic efficacy in traditional herbal formulations.

The WCR raw materials underwent quality control testing in accordance with the Thai Herbal Pharmacopoeia 2018 [7] (Table 3) to ensure consistency and safety. The ethanol extract content confirms the presence of ethanol-soluble, semi-polar phytochemicals, including flavonoids, alkaloids, and terpenoids, which are critical for product efficacy and standardization. The water extract content indicates the presence of water-soluble components, such as polysaccharides, glycosides, and phenolic compounds, thereby supporting product quality by verifying key active constituents. The moisture content remains well below the 10% threshold, confirming the suitability of the dried herbal material and reducing the risk of microbial growth, which contributes to product stability and shelf life [12,13]. The total ash content, representing organic and inorganic residues, is below the 10% limit [12,13], indicating high material purity and minimal contamination, which is essential for quality assurance. The acid-insoluble ash content quantifies ash, primarily silica, that does not dissolve in hydrochloric acid, serving as a key indicator of contamination from soil or other foreign particles and supporting quality control. The elevated acid-insoluble ash content, which exceeds the typical threshold of 2–3% [12,13], is attributed to the abundance of large macrosclereids characteristic of the *Smilax* genus and the presence of cystoliths, a key diagnostic feature of *Acanthus ilicifolius*. These results demonstrate that microscopic parameters are essential in pharmacognostic evaluation, as they connect anatomical features with physicochemical quality indices. Such characteristics not only account for variations in ash values but also enhance the credibility of microscopic examination as a vital method for herbal quality control and standardization. The measured constant values represent the collective influence of all herbal components in the formulation and serve as distinctive parameters specific to the WCR. The detailed specification is provided in the Supplementary Materials.

A significant challenge in assessing the quality of WCR arises from its preparation as a water extract. While this approach follows traditional clinical practice, it complicates analysis. Thin-layer chromatography (TLC) is commonly used for herbal authentication, but it is ineffective for highly polar, water-based extracts. In these cases, separation is poor, bands are faint or unresolved, and chromatograms are difficult to interpret, hindering reproducible quality control and increasing the risk of missing adulteration or variability. To address this, we used Compact Mass Spectrometry (CMS), which enables sensitive and rapid detection of chemical constituents in complex polar matrices. Replacing TLC with CMS provided more accurate and reliable chemical profiling of WCR, overcoming a key limitation in standardizing traditional water extracts. CMS analysis of WCR extracts (SIM mode, APCI positive) detected several compounds, including 1,8-cineole, α-amyrin, β-caryophyllene, naringenin, *trans*-*p*-coumaryl alcohol, *p*-coumaryl aldehyde, protocatechuic acid, stemone, stigmasterol, and stillbostemin D (Table 4, Figure 2). Of these, α-amyrin showed the highest ion intensity (2.4 × 10^6^), indicating that it is a prominent detectable compound under these conditions. However, since ion intensity does not directly reflect absolute concentration, these results should be considered qualitative evidence of compound presence rather than a measure of relative abundance. Additionally, “Kha” contributes to four compounds (1,8-cineole, β-caryophyllene, *trans*-*p*-coumaryl alcohol, and *p*-coumaryl aldehyde), indicating that it is a rich source of bioactive volatiles and phenolics. The use of SIM mode with APCI in positive ion mode enabled sensitive detection of trace components, as shown by the detection of compounds with low intensities (e.g., 8.4 × 10^4^ for stillbostemin D).

In addition to thin-layer chromatography (TLC) and high-performance thin-layer chromatography (HPTLC), high-performance liquid chromatography (HPLC) is widely employed to assess chemical quality. The chromatogram in Figure 3 displays multiple peaks, each corresponding to distinct phytochemicals present in the WCR extract. The main peaks appear at retention times of 12.578 and 20.005 min, indicating the presence of major compounds in WCR. Previous research [14] identified *trans*-*p*-coumaryl alcohol as a potential marker for WCR at 12.578 min. However, the major peak at approximately 20.005 min could not be conclusively identified. Although repeated chromatographic separation was attempted, the compound appeared to be chemically unstable during purification, leading to degradation and difficulty in obtaining a pure isolate for structural elucidation. Furthermore, some compounds detected by CMS may lack strong chromophores and therefore may not be clearly observed in UV-based HPLC detection. Therefore, definitive identification of other major peaks could not be achieved in the present study. Further investigation using LC-MS or HPLC-MS is warranted to enable definitive peak annotation and more comprehensive chemical characterization of WCR. HPLC is widely used to establish chemical fingerprints and monitor batch-to-batch consistency [15,16], yet its application in herbal specification presents notable limitations. A standard ultraviolet (UV) detector only detects compounds with chromophores, leaving non-chromophore constituents undetected. For water-based extracts such as WCR, the strong polarity and complexity of the matrix further complicate HPLC separation, often leading to overlapping peaks or weak UV signals. Additionally, HPLC requires expensive instrumentation, labor-intensive sample preparation, and specific detection conditions that may not be suitable for highly polar compounds. In contrast, Compact Mass Spectrometry (CMS) provides targeted phytochemical profiling, detecting several compounds absent from the HPLC chromatogram. The two previously reported biomarkers were also clearly observed in the CMS data, supporting the reliability of this method. These results underscore the challenges of relying exclusively on HPLC for the comprehensive specification of traditional water extracts. Consequently, this study employed CMS as an alternative and complementary technique, providing higher sensitivity, rapid detection, and improved suitability for profiling complex polar matrices [17]. Several limitations persist. Ion intensity measured by CMS does not directly correspond to quantitative abundance, and HPLC with UV detection may overlook constituents lacking chromophores. Quantitative analysis of marker compounds was not conducted and should be addressed in future studies. Nevertheless, HPLC remains a valuable fingerprinting tool, as its chromatographic patterns represent the overall chemical identity of the formula and facilitate batch-to-batch comparisons. Although HPLC alone may be insufficient for specification, it serves a critical complementary function in verifying the consistency and authenticity of WCR.

Beyond its function as a chemical marker, the biological significance of *trans*-*p*-coumaryl alcohol was examined by assessing its effect on nitric oxide (NO) production in an in vitro inflammatory model. Previous research demonstrated that *trans*-*p*-coumaryl alcohol inhibits NO production in lipopolysaccharide (LPS)-activated mouse peritoneal macrophages [18]. Consistently, the current study shows that *trans*-*p*-coumaryl alcohol significantly reduces NO production in LPS-stimulated RAW 264.7 macrophages without causing cytotoxicity at effective concentrations. The reduction in NO levels was concentration-dependent, indicating a direct pharmacological effect rather than an outcome of decreased cell viability. Nitric oxide is a critical inflammatory mediator produced by activated macrophages and plays a central role in the pathogenesis of inflammatory skin diseases such as psoriasis. Consequently, suppression of NO production is widely used as an in vitro indicator of anti-inflammatory potential [1,19]. The pronounced inhibitory effect of *trans*-*p*-coumaryl alcohol supports its role as a bioactive compound contributing to the anti-inflammatory properties of the WCR. Importantly, *trans*-*p*-coumaryl alcohol demonstrated greater NO inhibitory activity than indomethacin at equivalent concentrations, suggesting relatively high potency in this cellular model. This observation aligns with previous reports identifying *trans*-*p*-coumaryl alcohol as a bioactive constituent linked to the anti-inflammatory activity of WCR [14].

Indomethacin is a well-established nonsteroidal anti-inflammatory drug, but *trans*-*p* -coumaryl alcohol showed a stronger inhibitory effect, suggesting its potential as a natural anti-inflammatory agent. This study used an in vitro macrophage model so further research is needed to clarify the molecular mechanisms, including analyses of iNOS expression and upstream inflammatory pathways. In vivo studies and clinical validation are also necessary to confirm *trans*-*p*-coumaryl alcohol’s therapeutic relevance for psoriasis. In addition, the anti-inflammatory activity of the whole WCR extract was investigated in our earlier study [14], in which the formulation suppressed pro-inflammatory mediators in LPS-stimulated macrophages. However, this model represents preliminary evidence and does not fully reflect psoriasis-specific pathogenesis. Therefore, further validation using psoriasis-relevant models is still required.

Quantitative analysis revealed that the Wannachawee formulation contained 8.77 ± 0.14 mg/g extract of *trans*-*p*-coumaryl alcohol. This measurable presence of the phenylpropanoid aligns with previous studies identifying plant-derived phenolic constituents as significant contributors to anti-inflammatory activity [20].

*Trans*-*p*-coumaryl alcohol exhibited potent inhibitory activity against nitric oxide (NO) production, reducing NO levels to 17.16 ± 1.46% of the LPS-induced control at its highest non-cytotoxic concentration (6.25 µg/mL). Consequently, the quantified level of *trans*-*p*-coumaryl alcohol serves as both a suitable chemical marker for quality control and a potential bioactive marker associated with the extract’s anti-inflammatory potential. Additionally, the successful quantification using the validated HPLC method confirms the applicability of the developed analytical procedure for routine quality assessment of the Wannachawee formulation.

The developed HPLC method for determining *trans*-*p*-coumaryl alcohol satisfied the ICH Q2(R1) validation criteria. It exhibited strong specificity, with the analyte peak clearly separated from matrix components, ensuring accurate identification in complex samples. Linearity was demonstrated over the range of 1 to 20 µg/mL (R^2^ = 0.9992), indicating a strong correlation between peak area and concentration. Sensitivity was adequate, with limits of detection (LOD) and quantification (LOQ) of 0.64 µg/mL and 1.94 µg/mL, respectively, allowing for detection and quantification at low concentrations. Accuracy, evaluated through recovery studies, ranged from 101.30% to 108.60%, with relative standard deviations below 2%, confirming method reliability. Precision was also high, with intraday and interday relative standard deviations under 2%, indicating good repeatability and intermediate precision. These findings demonstrate that the method is reliable, accurate, precise, and suitable for routine quantitative analysis of *trans*-*p*-coumaryl alcohol in the formulation.

The integration of CMS profiling and HPLC fingerprinting may offer a practical toolkit for hospitals and manufacturers to authenticate raw materials, ensure batch-to-batch consistency, and establish quality control protocols. This strategy supports the clinical reliability of WCR and enhances its potential for commercialization and global acceptance.

## 4. Materials and Methods

### 4.1. Collection of Plant Material and Authentication

#### 4.1.1. Selection of Herbal Plants

All crude herbal plants used in WCR were sourced from Prapokklao Hospital in Chanthaburi Province, Thailand. The plant materials were identified by Wannaree Charoensup, a botanist at the Faculty of Pharmacy, Chiang Mai University, and deposited in the Herbarium of the Faculty of Pharmacy, Chiang Mai University, Chiang Mai, Thailand. Information on the herbal ingredients in the Wannachawee recipe and Voucher No. are shown in Table 8.

#### 4.1.2. Preparation of Herbal Samples

The crude drugs (see Supplementary Materials) were dried at 50 °C in a hot air oven (Scientific Promotion, Bangkok, Thailand) to remove moisture. All samples were weighed and then ground through a No. 60 sieve.

### 4.2. Determination of Macroscopic and Microscopic Characteristics

Plant materials were classified based on their sensory, macroscopic, and microscopic features. The identification process comprised three key steps: selecting representative materials, preparing slides or powders, and examining the distinguishing characteristics.

The herbal samples were microscopically examined with an Olympus CH2 microscope (Olympus Corporation, Tokyo, Japan) equipped with a camera lucida to enable precise recording and illustration of diagnostic microscopic features

### 4.3. Physical and Chemical Properties [7]

#### 4.3.1. Azeotropic Distillation Method

A mixture of toluene (200 mL) and water (2 mL) from RCI Labscan (V.S. CHEM HOUSE, Bangkok, Thailand) was added to a 500-mL round-bottom flask and subjected to distillation for approximately 2 h. After cooling to room temperature, the initial water volume (V1) was recorded. Herbal powder (10 g) was subsequently added, and the mixture was distilled at an initial rate of 2 drops per second, which was later increased to 4 drops per second. Distillation continued until the herbal water was completely extracted, followed by an additional 5 min of distillation. The receiving tube was cooled to room temperature, and any adhered water droplets were tapped down to ensure thorough mixing. The final water volume (V2) was recorded for subsequent calculation.

#### 4.3.2. Total Ash Content

A 3.0-g sample of herbal powder was placed in a crucible of known constant weight and incinerated in a high-temperature furnace (Carbolite Gero Ltd., Hope Valley, UK) at 450 °C until only non-carbon ash remained. The crucible was cooled in a desiccator (DURAN, DWK Life Sciences, Mainz, Germany) and weighed repeatedly until a constant mass was achieved. The total ash content was determined based on the final mass.

#### 4.3.3. Acid-Insoluble Ash Content

A crucible containing total ash at constant weight was treated with 25 mL of 2 M hydrochloric acid from RCI Labscan (V.S. CHEM HOUSE, Bangkok, Thailand) and heated on a water bath for 5 min. The resulting mixture was filtered through Whatman No. 41 filter paper (Cytiva, Marlborough, MA, USA), and the residue was washed with hot water until the filtrate reached a neutral pH. The filter paper with the residue was transferred to the original crucible and incinerated in a high-temperature furnace at 500 °C for 7 h. The crucible was cooled in a desiccator and weighed repeatedly until a constant weight was achieved. The acid-insoluble ash content was determined based on the final mass.

#### 4.3.4. Ethanol Extract Content

A 5.0-g sample was placed in a covered Erlenmeyer flask, and 100 mL of 95% ethanol (Liquor Distillery Organization, Excise Department, Bangkok, Thailand) was added. The mixture was shaken for 6 h and then left to stand for an additional 18 h. After a total extraction period of 24 h, the mixture was filtered using No. 1 filter paper (Cytiva, Marlborough, MA, USA). A 20-mL portion of the crude extract was evaporated to dryness on a water bath with gentle heating. The resulting residue was dried at 105 °C for 2 h, and then dried for an additional hour. After cooling in a desiccator, the sample was weighed repeatedly until a constant mass was achieved. The extractable matter content was then calculated.

#### 4.3.5. Water Extract Content

A 5.0-g sample of herbal powder was placed in a covered Erlenmeyer flask, and 100 mL of saturated chloroform-water was added. The mixture was shaken for 6 h and then allowed to stand for an additional 18 h. After a total extraction period of 24 h, the mixture was filtered using No. 1 filter paper. A 20-mL aliquot of the crude extract was evaporated to dryness in a water bath under gentle heating. The resulting residue was dried at 105 °C for 2 h, followed by an additional hour. After cooling in a desiccator, the sample was weighed repeatedly until a constant weight was achieved. The extractable matter content was subsequently calculated.

### 4.4. Extraction of WCR

Traditional extraction methods were employed, specifically boiling herbs in water. The coarse powder of WCR was decocted in water for 30 min. The resulting water extract was filtered and concentrated to a Brix (Atago Co., Ltd., Tokyo, Japan) value of 3–5, and then dried using a spray dryer (BUCHI, Bangkok, Thailand).

### 4.5. Identification by Compact Mass Spectrometry Method

The WCR extract solution (0.01 mg/mL in 99% methanol, HPLC-grade) was filtered through a 0.22 µm syringe filter. A 10 µL aliquot was injected into the compact mass spectrometer (CMS; Advion Inc., Ithaca, NY, USA). Mass spectral data were collected in selected ion monitoring (SIM) mode with a positive atmospheric pressure chemical ionization (APCI) source at a resolution of 0.5 to 0.7 *m*/*z* and compared to standards of rosmarinic acid (*m*/*z* 361.318 [M+H]+).

### 4.6. Chromatographic Fingerprints by High-Performance Liquid Chromatography (HPLC)

A Shimadzu Prominence High-Performance Liquid Chromatography (HPLC) system (SpectraLab Scientific Inc., ON, Canada) was utilized, comprising a DGU-20A3R degasser, two LC-20AD pumps, a SIL-20A autosampler, a CTO-20A column oven, and an SPD-20A UV-Visible detector. Chromatographic separation was achieved using a Mightysil RP-C18 GP column (250 × 4.6 mm, 5 µm) with a guard column. The mobile phase was a gradient of acetonitrile and formic acid in HPLC-grade water at a flow rate of 1.0 mL/min. The gradient program consisted of 10% acetonitrile, which was increased to 45% at 20 min, then to 50% at 40 to 45 min, and finally to 80%. Detection was performed at an absorption wavelength of 254 nm. The column temperature was set at 25 °C, and the injection volume was 20 µL [21].

### 4.7. Method Validation of HPLC [22]

#### 4.7.1. Specificity/Selectivity

Specificity was evaluated by comparing chromatograms obtained from the Wannachawee formulation (1 mg/mL), reference standard solution (10 µg/mL), and methanol (MeOH) as a blank. Each solution was injected at 20 µL under the established chromatographic conditions.

#### 4.7.2. Linearity and Range

The linearity of the method was assessed using *trans*-*p*-coumaryl alcohol (Toronto Research Chemicals, Toronto, ON, Canada) standard solutions prepared from a 1 mg/mL stock solution and diluted to five concentration levels (1–20 µg/mL). Each level was analyzed in triplicate. Calibration curves were constructed by plotting peak area against concentration, and linear regression analysis was performed to obtain the regression equation and coefficient of determination (R^2^).

#### 4.7.3. LOD and LOQ

The LOD and LOQ were estimated based on the standard deviation of the response (σ) at the lowest concentration level (1 µg/mL) and the slope (S) of the calibration curve, using the equations LOD = 3.3(σ/S) and LOQ = 10(σ/S). The σ value was determined from replicate measurements at this concentration level.

#### 4.7.4. Accuracy (Recovery Test)

The accuracy of the method was evaluated by a recovery study using the standard addition method. Known amounts of *trans*-*p*-coumaryl alcohol at three concentration levels (5, 12, and 20 µg/mL) were spiked into the sample solution (1 mg/mL). Each concentration level was analyzed in triplicate (*n* = 3). The percentage recovery was calculated using the calibration curve. The method was considered accurate if the recovery values were within 90–110% with a relative standard deviation (RSD) of less than 2%, in accordance with validation criteria based on analyte concentration levels reported by APVMA guidelines [23].

#### 4.7.5. Precision

- Repeatability (intra-day)

The intraday precision of the method was evaluated by analyzing *trans*-*p*-coumaryl alcohol at three concentration levels (5, 10, and 20 µg/mL). Each concentration was injected five times (*n* = 5) within the same day under identical analytical conditions. The precision was expressed as the percentage relative standard deviation (%RSD) of the measured responses. The method was considered precise if the %RSD values were less than 2%, in accordance with the acceptance criteria recommended by ICH Q2(R1).

- Intermediate precision (inter-day/analyst)

The interday precision (intermediate precision) of the method was evaluated over three consecutive days (Day 1, Day 2, and Day 3). *Trans*-*p*-coumaryl alcohol standard solutions at three concentration levels (5, 10, and 20 µg/mL) were analyzed in triplicate (*n* = 3) on each day. The precision was expressed as the percentage relative standard deviation (%RSD) of the measured responses across the three days. The method was considered to have acceptable interday precision if the %RSD values were less than 2%, in accordance with the acceptance criteria recommended by ICH Q2(R1).

### 4.8. Determination of Nitric Oxide (NO) Inhibitory Activity [24]

#### 4.8.1. Cell Proliferation Assay

The antiproliferative effect of *trans*-*p*-coumaryl alcohol on RAW 264.7 cells were evaluated using the SRB assay. Cells (5 × 10^4^ cells/well) were seeded in 96-well plates and cultured in DMEM (GIBCO™, Thermo Fisher Scientific, MA, USA) supplemented with 10% FBS (GIBCO™, Thermo Fisher Scientific, Waltham, MA, USA) and antibiotics (GIBCO™, Thermo Fisher Scientific, MA, USA) at 37 °C in a 5% CO_2_ incubator for 24 h. Cells were then treated with various concentrations of *trans*-*p*-coumaryl alcohol or indomethacin and incubated for a further 24 h. Cells were fixed with cold 10% TCA, stained with 0.057% SRB, and the bound dye was solubilized in Tris base solution. Absorbance was measured at 540 nm using a microplate reader. All treatments were performed in triplicate.

#### 4.8.2. Nitric Oxide Assay

The inhibitory effect of *trans*-*p*-coumaryl alcohol on nitric oxide (NO) production was evaluated in RAW 264.7 cells. Cells (5 × 10^4^ cells/well) were seeded in 96-well plates and cultured in DMEM supplemented with 10% (*v*/*v*) FBS, 100 U/mL penicillin, and 100 µg/mL streptomycin at 37 °C in a humidified 5% CO_2_ incubator for 24 h. Cells were then treated with various concentrations of *trans*-*p*-coumaryl alcohol or indomethacin (positive control) for 1 h, followed by stimulation with LPS (Sigma-Aldrich, St. Louis, MI, USA) 1 µg/mL and further incubation for 24 h. The culture supernatant was collected and the nitrite level, a stable metabolite of NO, was determined using Griess reagent. Absorbance was measured at 540 nm with a microplate reader.

### 4.9. Determination of trans-p-Coumaryl Alcohol in Samples

The *trans*-*p*-coumaryl alcohol content in the Wannachawee formulation was determined using the validated HPLC method. The sample was prepared by accurately weighing the formulation and dissolving it in a water-methanol (MeOH) mixture to a final concentration of 1 mg/mL. The solution was filtered through a 0.45 µm membrane filter prior to analysis. The prepared sample was injected into the HPLC system in triplicate (*n* = 3). The amount of *trans*-*p*-coumaryl alcohol in the sample was quantified based on the calibration curve constructed from the reference standard. The peak area obtained from the sample was applied to the calibration curve equation to determine the corresponding concentration of *trans*-*p*-coumaryl alcohol, which was then expressed as mean ± S.D. (*n* = 3). Additional details are provided in the Supplementary Materials.

## 5. Conclusions

This study established comprehensive quality control parameters and a chemical fingerprint for the Wannachawee Recipe (WCR), a traditional Thai herbal formulation for psoriasis. Microscopic analysis confirmed the presence and authenticity of all eight herbal components, supporting the reliability of WCR as a standardized product. Physicochemical testing, in accordance with Thai Herbal Pharmacopoeia standards, revealed the quality specifications of WCR. Compact mass spectrometry identified multiple bioactive compounds, while HPLC fingerprinting ensured batch-to-batch consistency. The validated analytical method demonstrated adequate specificity, precision, and accuracy, enabling reliable quantification of *trans*-*p*-coumaryl alcohol, a key bioactive marker of WCR (8.77 mg/g extract), for quality specification. Additionally, *trans*-*p*-coumaryl alcohol showed significant anti-inflammatory activity by inhibiting nitric oxide production in activated macrophages, with greater inhibitory effects than indomethacin.

Together, these methods provide a scientific foundation for the safety, efficacy, and reproducibility of WCR, supporting its recognition as a standardized Thai traditional medicine. The defined specifications and analytical profiles can be used by hospitals and traditional medicine centers to ensure authenticity and consistent quality in patient care. The validated data support official registration and promote broader recognition of Thai traditional medicine. By ensuring reproducible quality, safety, and efficacy, this research facilitates large-scale production, clinical integration, and potential export of standardized WCR products, thereby benefiting both patients and the Thai herbal medicine industry.

## Figures and Tables

**Table 1:** Microscopic characteristics observed in the Wannachawee Recipe (WCR) powder and their corresponding herbal sources.

No. (from Appendix Figure A9)	Diagnostic Feature Observed in WCR Powder	Probable Herbal Source in WCR	Matching Figure and No. (from Appendix Figure A1, Figure A2, Figure A3, Figure A4, Figure A5, Figure A6, Figure A7 and Figure A8)
1	Starch grains	*Alpinia galanga*, *Smilax glabra*, *Smilax corbularia*, *Smilax* sp., *Stemona collinsae*, *Acanthus ilicifolius*	Figure A1 (1), Figure A2 (1), Figure A3 (1), Figure A4 (1), Figure A6 (2), Figure A8 (5)
2	Cork	*Stemona involute*, *Stemona collinsae*	Figure A5 (2), Figure A6 (1)
3	Hypodermis containing greyish substances	*Stemona involuta*	Figure A5 (1)
4	Trichome	*Rhinacanthus nasutus*	Figure A7 (3)
5	Epidermis	*Alpinia galanga*, *Stemona involuta,* *Rhinacanthus nasutus,* *Acanthus ilicifolius*	Figure A1 (4), Figure A5 (1), Figure A7 (1), Figure A8 (1)
6	Parenchyma containing starch grains	*Alpinia galanga*, *Smilax glabra*, *Stemona collinsae*	Figure A1 (3), Figure A2 (3), Figure A6 (3)
7	Parenchyma containing starch grains and oil cells	*Alpinia galanga*	Figure A1 (2)
8	Parenchyma containing prismatic crystals	*Stemona collinsae*	Figure A6 (12)
9	Parenchyma containing rod crystals attached to fibers	*Acanthus ilicifolius*	Figure A8 (15)
10	Parenchyma containing needle-shaped crystals attached to large macrosclereids	*Smilax corbularia*, *Smilax* sp.	Figure A3 (6), Figure A4 (9)
11	Macrosclereids	*Smilax corbularia* *Smilax* sp., *Stemona involuta*, *Stemona collinsae,* *Acanthus ilicifolius*	Figure A3 (3), Figure A4 (4), Figure A5 (8), Figure A6 (5), Figure A8 (9)
12	Cell accumulants a. reddish-brown substance b. rod crystals c. transparent matter d. cystolith	*Acanthus ilicifolius,* *Smilax glabra*, *Acanthus ilicifolius,* *Stemona collinsae,* *Acanthus ilicifolius*	Figure A8 (11) Figure A2 (2), Figure A8 (15), Figure A5 (7), Figure A7 (6)
13	Fragments of fiber	*Stemona collinsae*, *Smilax* sp., *Acanthus ilicifolius*	Figure A6 (9), Figure A4 (5), Figure A8 (15)
14	Bundle of fiber and prism sheath	*Stemona collinsae*	Figure A6 (8)
15	Sclereids some containing rod crystals	*Smilax glabra*	Figure A2 (6)
16	Spiral vessels	*Alpinia galanga*, *Rhinacanthus nasutus*, *Acanthus ilicifolius*	Figure A1 (6), Figure A7 (7), Figure A8 (13)
17	Reticulated vessels	*Alpinia galanga*, *Smilax corbularia*, *Smilax* sp., *Rhinacanthus nasutus*, *Acanthus ilicifolius*	Figure A1 (8), Figure A3 (5), Figure A4 (5,8), Figure A7 (8,9), Figure A8 (14)
18	Scalariform vessels	*Alpinia galanga*, *Smilax corbularia*, *Smilax* sp.	Figure A1 (7,8), Figure A3 (5), Figure A4 (8)
19	Scalariform-bordered pitted vessels (associated with porous parenchyma)	*Smilax corbularia*, *Smilax* sp.	Figure A4 (5), Figure A4 (5,6,7,8)
20	Bordered pitted vessels	*Smilax glabra*, *Smilax corbularia*, *Smilax* sp., *Stemona involute*, *Stemona collinsae*, *Acanthus ilicifolius*	Figure A2 (4), Figure A3 (5), Figure A4 (8), Figure A5 (6), Figure A6 (11), Figure A8 (12)

**Table 2:** Microscopic diagnostic characteristics of 8 herbal components in WCR.

Sample in WCR	Characteristics	Appendix
Kha	Starch grains (1), parenchyma containing starch grains and oil cell (2), epidermis (4), parenchyma studded with silica crystalline matter (5)	Figure A1
Khao Yen Tai	Starch grains (1), bordered pitted vessel and parts of spiral vessel (4), sclereids (6)	Figure A2
Khao Yen Nuea	Starch grains (1), xylem elements showing reticulated vessels bordered pitted vessels and scalariform vessels (5), needle shape crystals (6)	Figure A3
Khao Yen Jeen	Starch grains (1), large scalariform-bordered pitted vessels (7), needle shape crystals (9)	Figure A4
Hua Ta Pead	Epidermis with under lying hypodermis containing greyish substances (1)	Figure A5
Non Tai Yak	Starch grains (2), transparent masses (7), bundle of fiber associated with parenchyma containing prismatic crystals (8), parenchyma containing prismatic crystals associated with thin-wall parenchyma (12)	Figure A6
Thong Pan Chang	Trichome (3)	Figure A7
Ngueak Plaamo	Cystolith (6), macrosclereid (9), reddish brown substances (11), fibers with some associated with parenchyma containing rod crystals (15)	Figure A8

**Table 3:** Physical and chemical properties of WCR.

NO.	Quality Assessment	Results of WCR
1	Ethanol extract content (% *w*/*w*)	8.7292 ± 0.15
2	Water extract content (% *w*/*w*)	18.8919 ± 0.09
3	Moisture content (% *v*/*w*)	8.1561 ± 0.06
4	Total ash content (% *w*/*w*)	8.5140 ± 0.03
5	Acid-insoluble ash content (% *w*/*w*)	3.2430 ± 0.04

**Table 4:** Molecular mass and intensity of the active ingredients found in the WCR extract using SIM mode (APCI in positive mode).

Chemical Compound	Molecular Mass [M]	Molecular Mass in Positive Ion [M+H]^+^	Intensity	WCR Extract	WCR Herb It Is Found in
1,8-cineole	154.249	155.249	3.6 × 10^5^	+	Kha
α-amyrin	119.380	120.380	2.4 × 10^6^	++	Ngueak Plaa Mo
ß-caryophyllene	204.357	205.357	8.7× 10^4^	+	Kha
Naringenin	272.068	273.068	1.2 × 10^5^	+	Khao Yen Nuea
*trans*-*p*-coumaryl alcohol (pure)	150.177	151.177	1.2 × 10^5^	+	Kha
*trans*-*p*-coumaryl aldehyde (pure)	148.158	149.158	1.6 × 10^5^	+	Kha
Protocatechuic acid	154.121	155.121	3.1 × 10^5^	+	Khao Yen Jeen
Stemone	143.227	144.227	4.1 × 10^5^	+	Non Tai Yak
Stigmasterol	412.702	413.702	1.2 × 10^5^	+	Ngueak Plaa Mo
Stillbostemin D	258.310	259.310	8.4 × 10^4^	+	Hua Ta Pead

++ = abundant (high peak intensity), + = detected (low to moderate peak intensity).

**Table 5:** Accuracy of *trans*-*p*-coumaryl alcohol determination (recovery study).

Level	Amount Added (µg/mL)	Amount Found (µg/mL)	%Recovery (*n* = 3, Mean ± S.D.)	%RSD
Low	5.00	5.43	108.60 ± 0.95	0.87
Mid	12.00	12.53	104.41 ± 1.52	1.46
High	20.00	20.26	101.30 ± 1.12	1.11

**Table 6:** Intraday Precision.

Concentration (µg/mL)	Replicate 1	Replicate 2	Replicate 3	Replicate 4	Replicate 5	Mean ± S.D.	%RSD
5	4.83	4.83	4.83	4.84	4.84	4.83 ± 0.01	0.11
10	9.47	9.46	9.42	9.53	9.53	9.48 ± 0.04	0.46
20	20.85	20.88	20.97	20.90	20.85	20.89 ± 0.05	0.22

**Table 7:** Interday Precision.

Day	Concentration (µg/mL)	Replicate 1	Replicate 2	Replicate 3	Mean ± S.D.	% RSD
Day 1	5	5.37	5.34	5.37	5.36 ± 0.01	0.23
	10	10.05	10.05	10.03	10.04 ± 0.01	0.07
	20	21.28	21.27	21.26	21.27 ± 0.01	0.05
Day 2	5	4.93	4.94	4.93	4.93 ± 0.01	0.12
	10	9.63	9.60	9.66	9.63 ± 0.02	0.25
	20	20.96	20.99	21.01	20.99 ± 0.02	0.10
Day 3	5	4.88	4.90	4.90	4.89 ± 0.01	0.12
	10	9.22	9.22	9.16	9.20 ± 0.03	0.31
	20	20.60	20.62	20.66	20.63 ± 0.02	0.11

**Table 8:** List of plants used in Wannachawee recipe.

Thai Name	Scientific Name	Family	Part Used	Voucher No.
Kha	*Alpinia galanga* (L.) Willd.	Zingiberaceae	Rhizome	0023406
Khao Yen Tai	*Smilax glabra* Wall.ex Roxb.	Smilacaceae	Rhizome	0023412
Khao Yen Nuea	*Smilax corbularia* Kunth.	Smilacaceae	Rhizome	0023411
Khao Yen Jeen	*Smilax* sp.	Smilacaceae	Rhizome	0023413
Hua Ta Pead	*Stemona involuta* Inthachub.	Stemonaceae	Root	0023409
Non Tai Yak	*Stemona collinsae* Craib.	Stemonaceae	Root	0023408
Thong Pan Chang	*Rhinacanthus nasutus* (L.) Kurz.	Acanthaceae	Aerial part	0023410
Ngueak Plaamo	*Acanthus ilicifolius* L.	Acanthaceae	Aerial part	0023407

## Data Availability

The data supporting this study are available in the article and Supplementary Materials.

## Supplementary Materials

The following supporting information can be downloaded at: https://www.mdpi.com/article/10.3390/plants15091344/s1.

## Abbreviations

The following abbreviations are used in this manuscript:

APCI	Atmospheric Pressure Chemical Ionization
CMS	Compact Mass Spectrometry
HPLC	High-Performance Liquid Chromatography
LOD	Limit of Detection
LOQ	Limit of Quantification
LPS	Lipopolysaccharide
NO	Nitric Oxide
RSD	Relative Standard Deviation
SIM	Selected Ion Monitoring
THP	Thai Herbal Pharmacopoeia
WCR	Wannachawee Recipe
