# Supplementary material for: Bridging Traditional Wisdom and Evidence-Based Pharmaceutics: Comprehensive Specification and Biological Activity of the Wannachawee Recipe for Psoriasis
Source: Plants (Basel). 2026 Apr 28;15(9):1344. doi: 10.3390/plants15091344 (PMC13165126; doi:10.3390/plants15091344)
Supplement: Supplementary file 1 [file plants-15-01344-s001.zip › plants-4239524-supplementary.pdf]

ตำรับยาวรรณฉวี, ยาผง

Wannachawee Recipe Powder

Ingredient Wannachawee Recipe Powder contains *Alpinia galanga* (Kha), *Smilax glabra* (Khao Yen Tai), *Smilax* sp. (Khao Yen Jeen), *Smilax corbularia* (Khao Yen Nuea), *Stemona involuta* (Hua Ta Pead), *Stemona collinsae* (Non Tai Yak), *Rhinacanthus nasutus* (Thong Pan Chang), and *Acanthus ilicifolius* (Ngueak Plaamoo).

Description A fine, dark-brown powder with a characteristic aromatic odor and a salty-bitter taste, obtained from spray-dried aqueous extract.

Identification

Macroscopic and microscopic identification of Wannachawee Recipe Powder is described in the main manuscript.

Chemical Analysis by High-Performance Liquid Chromatography (HPLC)

The main manuscript provides comprehensive details regarding the HPLC conditions and chromatographic analysis.

Reference standard solution

Trans-p-coumaryl alcohol in methanol

Quantitative determination of *trans-p*-coumaryl alcohol by HPLC

A calibration curve of *trans-p*-coumaryl alcohol is constructed using standard solutions at appropriate concentrations. The content of *trans-p*-coumaryl alcohol in the sample is calculated from the peak area using the external standard method. The content of *trans-p*-coumaryl alcohol in Wannachawee Recipe Powder, determined by HPLC, is not less than 8.0 mg/g of extract.

Requirements

***p*-Coumaryl alcohol content:** Not less than 8.0 mg/g extract

Moisture content: Not more than 10.0% w/w

Total ash: Not more than 10.0% w/w

Acid-insoluble ash: Not more than 3.5% w/w

Ethanol-soluble extractive: Not less than 8.5% w/w

Water-soluble extractive: Not less than 18.5% w/w

Microbial limit

Salmonella spp.: Not detected in 10 g or 10 mL of sample

Clostridium spp.: Not detected in 10 g or 10 mL of sample

Staphylococcus aureus: Not detected in 1 g or 1 mL of sample

Total viable count (oral use)

TAMC $\leq 10^3$ CFU/g or CFU/mL, not detected

TYMC $\leq 10^2$ CFU/g or CFU/mL, not detected

Heavy metals Limits (ppm):

Lead (Pb): Not more than 10 ppm

Cadmium (Cd): Not more than 0.3 ppm

Arsenic (As): Not more than 4 ppm

Mercury (Hg): Not more than 0.5 ppm

(According to Thai Herbal Pharmacopoeia, 2019)

Packaging and Storage: It shall be kept in well-closed containers, protected from light, and stored in a cool and dry place.
